# Antiplasmodial Activity and In Vivo Bio-Distribution of Chloroquine Molecules Released with a 4-(4-Ethynylphenyl)-Triazole Moiety from Organometallo-Cobalamins

**DOI:** 10.3390/molecules24122310

**Published:** 2019-06-21

**Authors:** Jeremie Rossier, Sara Nasiri Sovari, Aleksandar Pavic, Sandra Vojnovic, Tameryn Stringer, Sarah Bättig, Gregory S. Smith, Jasmina Nikodinovic-Runic, Fabio Zobi

**Affiliations:** 1 Department of Chemistry, University of Fribourg, Chemin du Musée 10, 1700 Fribourg, Switzerland; jeremie.rossier@unifr.ch (J.R.); sara.nasirisovari@unifr.ch (S.N.S.); sarah.baettig@unifr.ch (S.B.); 2 Institute of Molecular Genetics and Genetic Engineering, University of Belgrade, Vojvode Stepe 444a, 11000 Belgrade, Republic of Serbia; pavicaleksandarr@gmail.com (A.P.); sanvojnov@gmail.com (S.V.); 3 Department of Chemistry, University of Cape Town, Rondebosch 7701, South Africa; STRTAM001@myuct.ac.za (T.S.); gregory.smith@uct.ac.za (G.S.S.)

**Keywords:** antimalarial, prodrug, chloroquine, triazole, cobalamin, in vivo, zebrafish model

## Abstract

We have explored the possibility of using organometallic derivatives of cobalamin as a scaffold for the delivery of the same antimalarial drug to both erythro- and hepatocytes. This hybrid molecule approach, intended as a possible tool for the development of multi-stage antimalarial agents, pivots on the preparation of azide-functionalized drugs which, after coupling to the vitamin, are released with a 4-(4-ethynylphenyl)-triazole functionality. Three chloroquine and one imidazolopiperazine derivative (based on the KAF156 structure) were selected as model drugs. One hybrid chloroquine conjugate was extensively studied via fluorescent labelling for in vitro and in vivo bio-distribution studies and gave proof-of-concept for the design. It showed no toxicity in vivo (zebrafish model) as well as no hepatotoxicity, no cardiotoxicity or developmental toxicity of the embryos. All 4-(4-ethynylphenyl)-triazole derivatives of chloroquine were equally active against chloroquine-resistant (CQR) and chloroquine-sensitive (CQS) *Plasmodium falciparum* strains.

## 1. Introduction

Malaria is considered to be one of the most life-threatening and globally infectious diseases caused by a single-cell parasite called *Plasmodium* (including *P. falciparum*, *P. vivax*, *P. ovale*, *P. malariae*). This disease has remained one of the leading causes of morbidity and mortality over the past centuries throughout the world. According to the World Health Organization (WHO) in 2016, there have been about 216 million cases of malaria and 445,000 deaths worldwide [1]. A decrease of 4% annually in the number of malaria victims over the past decade was observed, and clinical malaria cases have declined from 2000 to 2015 in endemic parts of Africa. However, *P. falciparum*, the most lethal malaria species in humans, has become resistant to the most conventional antimalarial treatments, in turn resulting in a worldwide concern and the call for new strategies, and drugs that can act at different stages of the parasite life cycle [2,3,4].

**Figure 1:**
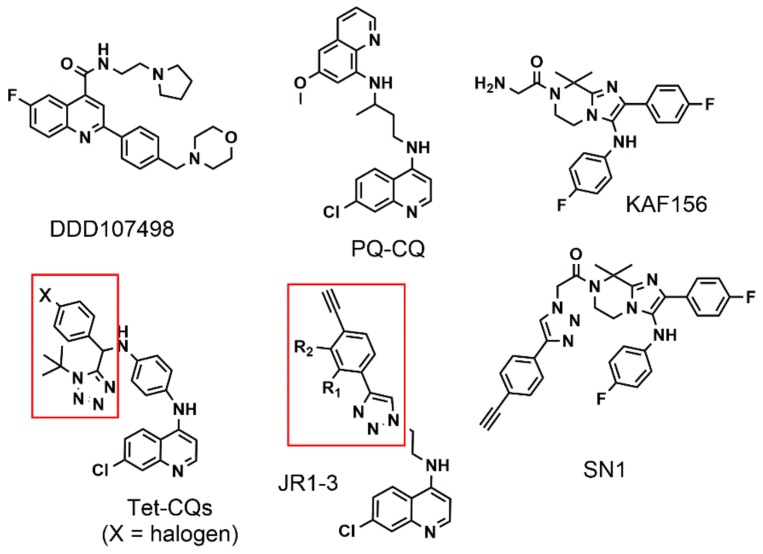
Molecular structures of antimalarial drugs mentioned in the introduction. JR1-3 and SN1 are the molecules designed for this study (vide infra for details). The red box indicates the structural feature that we took as reference for the design of the compounds.

New drug candidates are being identified as a result of the combined efforts of a global consortium (known as the NGBS (The NGBS consortium in malaria drug discovery is a collaboration between the Novartis Institute for Tropical Diseases (NITD), the Genomics Institute of the Novartis Research Foundation (GNF), the Biomedical Primate Research Centre (BPRC) and the Swiss Tropical and Public Health Institute (SwissTPH)) consortium) with the help of industry and private-public partnerships and by leading pharmaceutical companies (like Novartis or GlaxoSmithKline) that have the capacity for high-throughput screening of thousands of compounds [5]. These efforts have led to the chemical optimization of DDD107498 (Figure 1), a compound with a potent antimalarial activity against multiple life-cycle stages of the *Plasmodium* parasite and whose molecular target is the translation elongation factor 2 (eEF2), essential for parasite protein synthesis [6,7,8]. Other strategies being pursued involve the preparation of hybrid molecules to augment the activity profile of older drugs like chloroquine (CQ) [9]. In this approach, two different drug moieties, each with its own medicinal properties, are covalently linked in a single hybrid molecule, which presents a dual activity. The primaquine-chloroquine (PQ-CQ, Figure 1) hybrid reported by Lödige et al. exemplifies this approach [10]. The molecule displays in vitro activity against both asexual and sexual *P. falciparum* blood stages as well as *P. berghei* sporozoites and liver stages, and in vivo it is active against *P. berghei* liver and blood stages. 

Within this context, we decided to explore the possibility of using organometallic derivatives of cobalamin as a vector for antimalarial drugs as they were previously exploited for the targeted release of anticancer drugs [11,12,13]. For our study, we decided to evaluate triazole-containing chloroquinoline drugs, derivatized at the *N*-alkyl amino side chain and one imidazolopiperazine derivative based on the KAF156 (Ganaplacide) structure (Figure 1) [14]. The latter was selected as it is a rare example of drugs effective at the liver stage. The design of the CQ molecules was developed starting from the reference tetrazole embedded CQ derivatives prepared by Pandey et al. (Tet-CQs in Figure 1) [15]. These derivatives are active in animal models showing between ca. 60 and 99.99% suppression of parasitaemia on the fourth day of treatment. 

In our design, the CQ ring was left untouched, as this moiety is essential for the antimalarial activity of this class of compounds. Modifications of the CQ ring alter the p*K*a values of the whole molecule thereby affecting drug uptake by the parasite digestive vacuoles and its influx/efflux [16]. Modification at *N*-alkyl amino side chain are much less detrimental for the antimalarial activity the compounds [17]. Azoles were chosen because they are an important class of nitrogen containing heterocyclic compounds that often improve the therapeutic and biological activity of organic drugs. Their addition to the side chain of 7-chloro-4-aminoquinoline was suggested as a useful strategy for the design and development of new antimalarial agents [18]. Furthermore, tetrazole embedded CQ derivatives inhibit hemozoin possibly via heme complexation through the azole functionality [15] and, finally, triazole can be readily prepared via the Cu(I)-catalyzed azide-alkyne cycloaddition, a synthetic approach applicable to vitamin B_12_ chemistry [19].

The use of cobalamin (B_12_, Cbl) as a vector of antimalarial drugs for both the hepatocytic and erythrocytic stages rests on the consideration that vitamin B_12_ is transported and stored in the liver and that naturally occurring cobalamins have antimalarial activity of their own with IC_50_ values (blood stage) ranging from ca. 2–130 μM [20]. As such, they might act synergistically with other antimalarial compounds. Among the naturally occurring Cbls, Ado-Cbl (adenosylcobalamin) is the most active, followed by H_2_O-, CH_3_- and CN-Cbl. Furthermore, with the exception of CN-Cbl, all derivatives are approximately forty times more effective than chloroquine in inhibiting hemozoin formation [20]. Erythrocyte uptake ca. 20–50 pg B_12_/mL (ca. 0.1–0.25 μg/5 L human blood) [21]. The uptake depends on metabolic factors, it increases with rising reticulocyte count [22] and B_12_ content in red blood cells increases with levels of holoTCII (B_12_-bound transcobalamin II protein complex recognized by cell for B_12_ uptake) [23]. Finally, *P. falciparum* requires nanomolar concentrations of B_12_ for optimal growth as a cofactor for methionine synthase [24], the only B_12_-dependant enzyme in the intra-erythrocytic *P. falciparum* [25]. Chemaly has suggested that infected erythrocytes may uptake cobalamin while still bound to transcobalamin II or possibly haptocorrin [20], because the parasite is known to reactivate dormant endogenous red blood cell transporters, increase the permeability of the erythrocytic membrane [26,27], and infected erythrocytes are able to incorporate molecules as large as transferrin (ca. 80 kDa) [28]. 

## 2. Results and Discussion

### 2.1. Synthesis and Characterization

**Figure 2:**
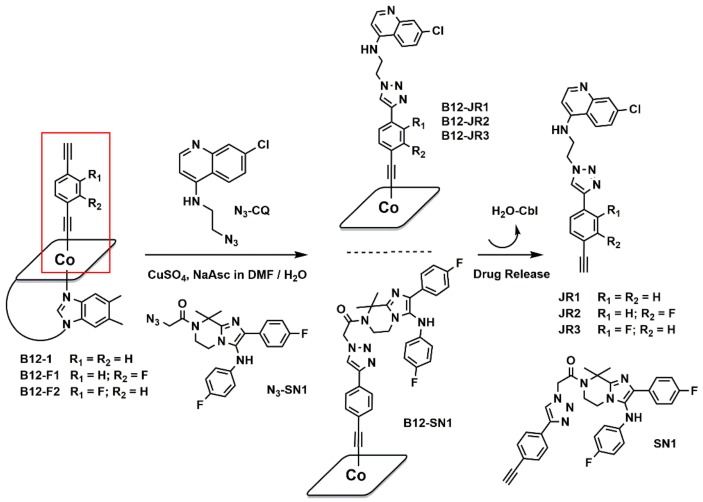
General synthetic scheme for the synthesis of derivatives B_12_-**JR1**-**JR3** and B_12_-**SN1**.

Vitamin B_12_ (B_12,_ Cbl) derivatives bearing antimalarial compounds were prepared by attaching releasable drugs at the cobalt ion via the synthetic protocol illustrated in Figure 2. The cyanide ligand of Cbl was substituted by either 1,4-diethynylbenzene [29] or 1,4-diethynyl-2-fluorobenzene to give cobalamine-1,4-diethynylbenzene (B_12-_**1**) or the fluorinated cobalamine-1,4-diethynyl-fluorobenzene intermediates B_12_-**F1** and -**F2** [11,30]. Following these reactions, an absorbance at 2117 cm^−1^ in the IR spectrum of the species replaced the original υC≡N frequency at 2134 cm^−1^ and was assigned to the cobalt-bound alkyne function. Concomitantly, the 4-diethylamino-1-methylbutylamino chain appended at the quinoline core structure of chloroquine was replaced by a 2-azidoethyl chain (to give *N*-(2-azidoethyl)-7-chloroquinolin-4-amine, N_3_-CQ) [31]. Similarly the primary amine of KAF156 was modified to a 2-azido-acetamide function giving 2-azido-1-(2-(4-fluorophenyl)-3-((4-fluorophenyl)amino)-8,8-dimethyl-5,6-dihydroimidazo[1–a]pyrazin-7(8*H*)-yl)ethan-1-one (N_3_-**SN1**, Figure 2) by using 2-azido-1,3-dimethylimidazolinium hexafluorophosphate and triethylamne in actetonitrile. This approach rendered the molecular units suitable for fusion via the Cu(I)-catalyzed azide-alkyne cycloaddition. Overall, following HPLC purification, the cycloaddition reaction gave vitamin B_12_ derivatives (B_12_-**JR1**, **2** and **3** and B_12_-**SN1**, Figure 2) in good yields (60–80%).

**Figure 3:**
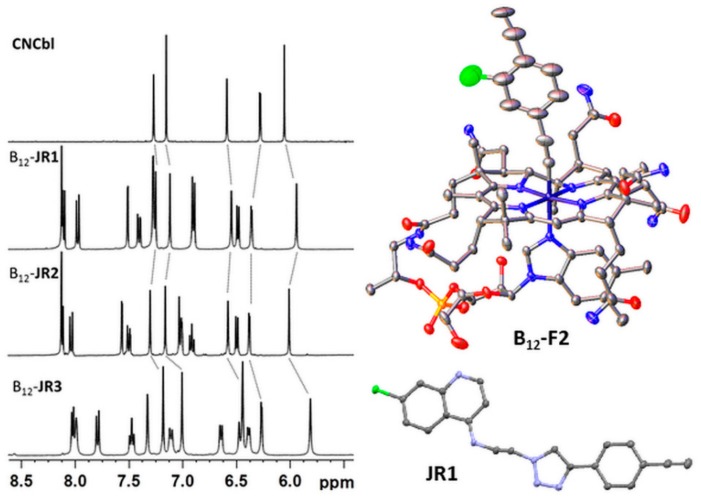
Left, aromatic region of the ^1^H-NMR spectra (D_2_O, 500 MHz) of CN-Cbl and of the derivatives B_12_-**JR1** to -**JR3**. Right, crystal structure of compound B_12_-**F2** and of **JR1** (thermal ellipsoids are shown at the 50% probability).

The aromatic regions of the ^1^H-NMR spectra of derivatives B_12_-**JR1-3** are shown in Figure 3 (full spectra of all molecules are found in ESI, Figures S1–S11). NMR was used with other techniques to assess the successful synthesis of the molecules. As evident in Figure 3, the aromatic region shows an increasing number of peaks after each modification which are related and consistent with the addition of the alkynylated linker and the CQ drug on cobalamin (see also Figures S4–S8 in ESI). As expected, with respect to free vitamin B_12_, most affected are the Co-bound dimethylbenzimidazole peaks and the ribose signals (in the 4.5–5 ppm regions, see Figures S4–S8 in ESI) which suffer an upfield shift after each cobalamin modification. From a stability point of view, all water-soluble compounds appeared photo stable in solution for at least seven days. Alongside the preparation of the vitamin B_12_ derivatives, ethynylphenyl-triazolyl-chloroquine and imidazolopiperazine drugs (**JR1-3** and **SN1**, Figure 2) were synthetized separately from the reaction of N3-CQ or N3-SN1 with either 1,4-diethynylbenzene (JR1, SN1) or 1,4-diethynyl-2-fluorobenzene (JR2 and JR3). The compounds were purified via HPLC and isolated as TFA salts. Of the series of compounds described above, we were able to obtain single crystals suitable for X-ray analysis of the B12-F2 precursor and the JR1 compound (namely, 7-chloro-*N*-(2-(4-(4-ethynylphenyl)-1*H*-1,2,3-triazol-1-yl)ethyl)quinolin-4-amine, Figure 3). B12-F2 crystalized in the orthorhombic space group P2_1_2_1_2_1_ (common for Cbl derivatives). Interestingly, the 1,4-diethynyl-2-fluorobenzene unit is found in two different conformations in the crystal structure. In both conformations, the Co-C≡C angle is bent (167.92° (2) and 173.27 (3)°) and the triple bond length is longer than in organic alkynes (1.211 (16) Å and 1.192 (17) Å respectively), which is consistent with previously reported acetylide cobalamins [30,32]. Compound JR1 crystalized in the P-1triclinic space group and it is also shown in Figure 3.

### 2.2. In Vitro Bio-Distribution and Antiplasmodial Activity

**Figure 4:**
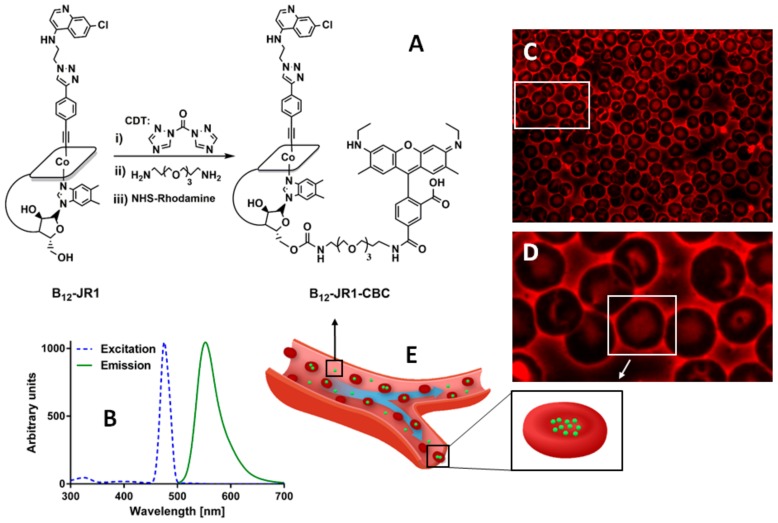
(**A**) Reaction steps leading to the synthesis of the green emitting B_12_-**JR1**-**CBC**. Conditions: (i) CDT, 12 h, DMSO; (ii) PEG, 12 h, anhydrous DMF; (iii) NHS-rhodamine, 12 h, TEA, anhydrous DMF. (**B**) Normalized excitation and emission profile of B_12_-**JR1**-**CBC** in a 1:1 H_2_O/MeOH solution. (**C**,**D**) Fluorescent microscope images of erythrocytes within full canine blood smear incubated with B_12_-**JR1**-**CBC** at 37 °C (GFP filter; 100 × magnification). (**E**) Conceptual drawing of B_12_-**JR1**-**CBC** accumulation within erythrocytes. **Note**: as a full blood smear was made, fluorescence around red blood cells is due to serum and/or burst erythrocytes.

Given that the pathogenesis of the disease is based on the rosetting phenomenon [33], occurring during the life cycle of the *P. falciparum* in red blood cells (RBCs) promoting the binding of parasitized RBCs to healthy ones, our conceptual design pivots on the possibility that when attached to the cobalamin scaffold antimalarial drugs can be delivered to both erythrocytes and hepatocytes and intracellularly released following the natural Cbl pathway. We therefore needed to establish if the modified vitamins could still accumulate within red blood cells to some extent. To this end, B_12_-**JR1** was modified at the sugar moiety with a fluorescent dye in order to track its bio-distribution both in vitro and in vivo [34]. As shown in the synthetic protocol illustrated in Figure 4, the 5′-OH group of the Cbl ribose was activated with 1,1′-carbonyl-di-(1,2,4-triazole) (CDT) and then reacted with an amine-terminated Polyethylene glycol (PEG) chain to promote amide bond formation [34]. This intermediate was then reacted with 5-carboxy-tetramethyl-rhodamine succinimidyl ester (NHS-Rhodamine) to yield the fluorescent B_12_-**JR1**-**CBC** molecule. B_12_-**JR1**-**CBC** was then incubated within full canine blood at 37 °C for 24 h in dark. After this period of time, red fluorescence was observed within erythrocytes in the full blood smear of the blood sample (Figure 4 C–D), and we could determine that between 11–13% of the compound associates with washed erythrocytes after 24 h incubation (Figure S21).

Having established the intracellular erythrocyte accumulation of B_12_-**JR1**-**CBC**, the B_12_ complexes B_12_-**JR1–3**, -**SN1** and their corresponding 4-(4-ethynylphenyl)-triazole drugs **JR1–3** and **SN1** were tested for their antiplasmodial activity against the erythrocyte stage of *Plasmodium falciparum*. All the compounds were screened against the NF54 CQS (chloroquine-sensitive) strain of *P. falciparum* and the multidrug-resistant *P. falciparum* Thailand K1 strain CQR (chloroquine-resistant) isolate. Table 1 presents the data obtained from this study.

Triazole-containing quinoline **JR1**, **JR2** and **JR3** showed good activity in the sensitive strain, while the imidazolopiperazine derivatives **SN1** and B_12_-**SN1** were inactive. B_12_-**JR-3** exhibited moderate activity in the NF54 strain of *P. falciparum* with IC_50_ values ca. 30–40 times higher than those of their corresponding drugs **JR1-3**. The decrease in activity of the vitamin B_12_ derivatives, compared to their corresponding free drugs, indicates that there are no synergistic effects between the molecular components (i.e., B_12_ and **JR#**). Indeed, the relatively low in vitro activity of the B_12_ derivatives could be attributed to low accumulation of these complexes in the digestive vacuole (DV) of the parasite as some cobalamin compounds are less likely to accumulate in DV due to limited pH trapping [20]. Nevertheless, **JR1-3**, B_12_-**JR1** and B_12_-**JR2** are equally active against the chloroquine-resistant (CQR) K1 strain of *P. falciparum*, with resistance indices very close to unity. In terms of their cytotoxicity, molecules B_12_-**JR1-3** showed moderate activity against human lung fibroblast MRC5 (IC_50_ > 70 μM) which brings their selectivity index to the value of 10 and higher. Furthermore, B_12_-**JR1** was screened against the Chinese Hamster Ovarian (CHO) cell-line at a concentration ca. 400-fold its NF54 IC_50_ value and was found not to be toxic against mammalian cells at this dose. This confirms that these B_12_ complexes are selective towards the malaria parasite and because the compounds retain their activity in the resistant strain show no cross-resistance.

### 2.3. Drugs Interaction with Monomeric Ferriprotoporphyrin IX (PPIX)

Chloroquine (CQ) exerts its antimalarial function by inhibiting hemozoin formation during the critical ferriheme detoxification process of the *Plasmodium* parasite [35,36,37,38,39]. The molecule acts either by: (a) directly complexing monomeric PPIX preventing its incorporation into the growing hemozoin crystals [36,40,41,42], (b) capping the hemozoin crystal [43,44], (c) binding to PPIX via π–π interactions between the its aromatic ring and the porphyrin ring [45,46], (d) shifting the haematin/μ-oxo PPIX dimer equilibrium [40], or (e) docking onto the fastest growing crystal face [47]. To obtain an insight into the possible mode of action of the 4-(4-ethynylphenyl)-triazole functionalized drugs, we decided to study their interaction with strictly monomeric ferriprotoporphyrin IX via the procedure established by Egan et al. [48,49].

**Figure 5:**
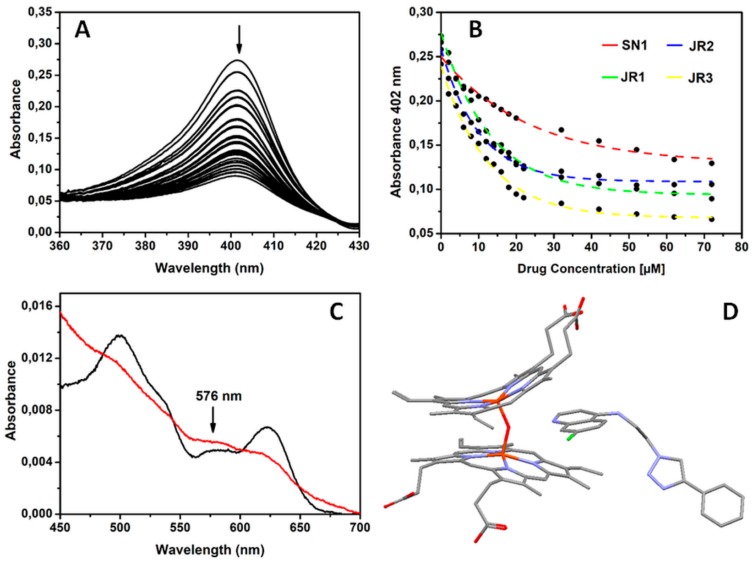
(**A**) Spectroscopic change observed in the Soret band of PPIX when it is titrated with drugs presented in this study. (**B**) Variation in absorbance of PPIX at 402 nm as a function of drug concentration. (**C**) Visible spectra of PPIX in 40% aqueous DMSO, pH 7.5 before (black line) and after addition of excess drug (red line). The spectrum of the latter is identical to that of the μ-oxo dimer previously reported. Note the peak maximum at 576 nm. (**D**) Density Functional Theory (DFT) optimized structure (gas-phase) of the interaction of a protonated 4-(4-ethynylphenyl)-triazole functionalized quinoline drug model with ferriprotoporphyrin IX μ-oxo dimer.

Accordingly, we performed spectrophotometric titrations of the drugs into a solution of PPIX and monitored the absorbance of the Soret band at 402 nm. Figure 5 shows the spectral changes observed in these experiments. The decrease in absorbance of the Soret band is either indicative of a drug-induced aggregation of PPIX, formation of π–π (donor acceptor) complexes or the changes reflect drug binding to PPIX [48,49]. Our molecular design offers at least four binding possibilities to ferriheme, but we were able to establish via DFT calculations that only the quinoline and the central triazole nitrogens are free of steric hindrance to support PPIX coordination (Figure S12 and S13). However, analysis of the spectrophotometric changes revealed that: a) none of our drugs are likely forming a 1:1 or 2:1 binding complex with PPIX as data do not fit the models of Egan et al.; b) the changes are dissimilar from those observed upon PPIX aggregation in aqueous solution [48]; c) the end-point spectra, after addition of a large excess of drugs, are identical to that of the μ-oxo dimer previously reported (Figure 5C) [50]. We conclude, therefore, that **JR1-3** might exert their antiplasmodial activity by promoting ferriprotoporphyrin μ-oxo dimer formation, thereby shifting the haematin/μ-oxo PPIX dimer equilibrium [40]. 

### 2.4. In Vivo Toxicity and Bio-Distribution

**Figure 6:**
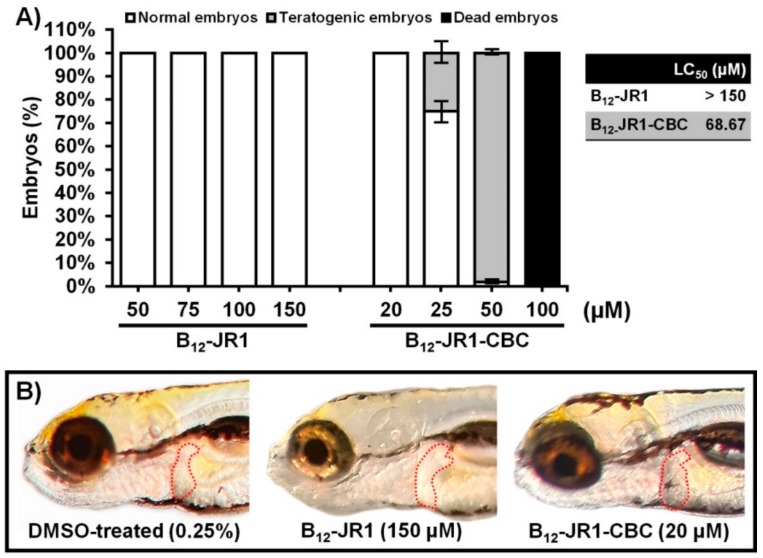
Toxicity evaluation of B_12_-**JR1** and B_12_-**JR1-CBC** in the zebrafish model over a period from 6 to 120 hpf, expressed as the LC_50_ values. Shown are data relative to (**A**) embryos survival/teratogenicity and (**B**) cardiotoxicity. The liver is outlined with a dashed line.

Based on the antiplasmodial activity and in vitro cytotoxicity results, B_12_-**JR1** was selected for further toxicity and distribution assessment in the zebrafish (*Danio rerio*) model, which proved a useful high-throughput model for determination of the acute toxicity in drug development efforts [51]. Toxicity assessment performed in the zebrafish model (nontransgenic, wild type embryos) during a period from 6 to 120 h post fertilization (hpf) showed that vitamin B_12_ (data not shown) and B_12_-**JR1** are neither toxic (Figure 6A) nor cardiotoxic (Figure 6B) at the doses up to 150 µM. On the other hand, the CBC-labelled molecule (B_12_-**JR1**-**CBC**) caused the appearance of pericardial edema and the whole-body edema/disintegration in 25% of embryos already at a dose of 25 µM (the same side effects were observed at 50 µM, in almost 100% of treated embryos). Notably, the toxicity of B_12_-**JR1** appeared to be markedly higher in vitro than in vivo (IC_50_ of 70 µM vs LC_50_ > 150 µM, respectively). According to the antiplasmodial activity and the in vivo toxicity results, B_12_-**JR1** has a large therapeutic window—higher than 24 for the NF54 strain (CQ-sensitive) and 16 for the K1 strain (CQ-resistant) of *P. falciparum* (Table 2).

**Figure 7:**
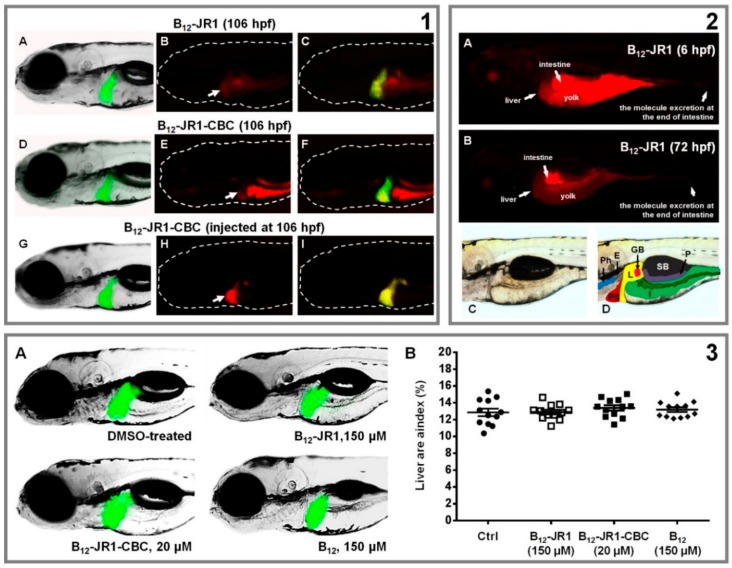
Bio-distribution and toxicity evaluation of B_12_-**JR1** and B_12_-**JR1-CBC** in the 120-hpf old transgenic *Tg*(*fabp10*:EGFP) zebrafish embryo with fluorescently labelled liver. **Panel 1** (top left). Top: accumulation of B_12_-**JR1** (150 µM) in the liver applied at 106 hpf in the embryo water. (A) Embryo imaged by a fluorescent microscope with a filter enabling only EGFP-labelled liver visualization. (B) Embryo imaged with a filter enabling B_12_-**JR1** visualization, but not EGFP-labelled liver. (C) Merged images A and B. Middle: accumulation of B_12_-**JR1-CBC** (20 µM) in the liver applied at 106 hpf in the embryo water. (D) Embryo imaged upon a filter enabling only EGFP-labelled liver visualization. (E) Embryo imaged with a filter enabling only B_12_-**JR1** visualization, and (F) merged *Tg*(*fabp10*:EGFP) and B_12_-**JR1-CBC** fluorescent signals in the same embryo. Bottom: an accumulation of B_12_-**JR1-CBC** (47.8 µg per an embryo, corresponding to 20 µM dose) in the liver microinjected (parenteral use) into embryo’s circulation at 106 hpf. Arrows in the panel 1 indicate the liver position within embryos body. **Panel 2** (top right): distribution of B_12_-**JR1** (150 µM) within the 120-hpf old embryos when applied at 6 hpf (the liver-free stage, A) or 72 hpf (the stage with functional liver, B). Lateral views (C and D) of the 120-hpf embryo with an overlay. Overlay outlines the pharynx (Ph), esophagus (E), liver (L), gallbladder (G), pancreas (P), swimmbladder (SB), and intestine (I). * marks intestinal lumen. **Panel 3** (bottom). Hepatotoxicity evaluation in the transgenic *Tg*(*fabp10*:EGFP) zebrafish embryos with EGFP-labelled liver after the embryos exposure to the tested compounds in a period from 72–120 hpf (A). The liver area index was assessed in 120-h old zebrafish embryos (B) and indicated no changes in the treated groups compared to the DMSO-treated (control) one (*n* = 15).

To determine the distribution and the accumulation of B_12_-**JR1** and B_12_-**JR1-CBC** through inner organs, transgenic *Tg*(*fabp10*:EGFP) zebrafish embryos with enhanced green fluorescent protein (EGFP)-labelled liver were exposed to the tested compounds in a period from 106–120 hpf (the developmental stage when the liver is highly vascularized and metabolically active). The results showed the preferential accumulation of both compounds in the liver of treated fish when applied in the water containing the embryos (Figure S23), as well as in the intestine due to the compounds absorption through mouth and skin (Figure 7, Panel 1). Longer exposure (from 72–120 hpf) of zebrafish embryos to the tested molecules resulted in higher drug accumulation in the liver, as evaluated by the fluorescence intensity (Figure S23). Moreover, when B_12_-**JR1** and B_12_-**JR1-CBC** were applied parenteral by the microinjection into the blood circulation, the compounds were solely detected in the liver of injected embryos, confirming thus their preferential accumulation in this organ. Importantly, after this period of exposure the compounds did not affect the liver development. Because liver toxicity presents one of the most common drawbacks of the drugs approved for the human use, the drugs synthesized in this study were examined for the possible hepatotoxicity in vivo. The transgenic *Tg*(*fabp*:EGFP) zebrafish embryos exposed at 72 hpf (a stage when the liver is becoming vascularized and performing metabolic transformation of the absorbed compounds) to the synthesized drugs were evaluated for the liver colour (a possible necrosis sign), size, fluorescence and the liver area index (the body area/liver area ratio). These are all parameters commonly used for hepatotoxicity evaluation and to follow the effect of applied therapies on the liver function.

The results show that both compounds, applied at the 150 µM for B_12_-**JR1** and 20 µM for B_12_-**JR1-CBC**, caused no changes in the liver size and fluorescence (Figure 7, Panel 3), and did not affect the liver area index. In addition, liver colour and yolk consumption in the treated fish, as additional specific phenotypic endpoints of hepatotoxicity, did not change in comparison to that of the control embryos (Figure 7, Panel 2), even when the embryos were exposed earlier at the 6 hpf stage (Figure 6B). Overall, the results indicate that B_12_-**JR1** is not hepatotoxic, at least, at a dose 16–24 higher than the one active against *Plasmodium* strains. Taken together, the results indicate that B_12_-**JR1** and B_12_-**JR1-CBC** indeed accumulate in the liver, and cause no hepatotoxicity, no cardiotoxicity or developmental toxicity at concentrations efficient against *P. falciparum*. This is of particular relevance in the therapy against the CQ-resistant malarial strains, since chloroquine, the most frequently used antiplasmodial drug, is very hepatotoxic already at 30 µM [52], while B_12_-**JR1** is not at 150 µM.

## 3. Materials and Methods

### 3.1. General Experimental Details

All chemicals were purchased from Sigma-Aldrich (St Louis, MO, USA) and used without further purification. HPLC analyses were performed on a Merck-Hitachi L7000 (Hitachi, Ltd., Tokyo, Japan). The analytical separations were conducted on a Macherey–Nagel Nucleodur PolarTec column (Macherey–Nagel AG, Oensingen, Switzerland, 5 μm particle size, 110 Å pore size, 250 × 3 mm). The preparative separations were conducted on a Macherey–Nagel Nucleodur C18 HTec column (Macherey–Nagel AG, Oensingen, Switzerland, 5 μm particle size, 110 Å pore size, 250 × 21 mm). HPLC solvents were for the system 1: aqueous trifluoroacetic acid 0.1% (A) and THF (B). HPLC solvents were for the system 2: aqueous trifluoroacetic acid 0.1% (A) and methanol (B). Using system 1, the compounds were separated using the following gradient: 0–5 min (75% solvent A), 5–35 (75% solvent A → 35% solvent A), 35–45 min (100% solvent B). Using system 2, the compounds were separated using the following gradient: 0–5 min (75% solvent A), 5–35 (75% solvent A → 0% solvent A), 35–45 min (100% solvent B). The flow rate was set to 0.5 mL/min for analytical separations and 5 mL/min for the preparative ones. The eluting bands were detected at 320 nm. The preparative plates used for the separation of the compounds 7-chloro-*N*-(2-(4-(4-ethynyl-3-fluorophenyl)-1*H*-1,2,3-triazol-1-yl)ethyl)quinolin-4-amine (**JR2**) and 7-chloro-*N*-(2-(4-(4-ethynyl-2-fluorophenyl)-1*H*-1,2,3-triazol-1-yl)ethyl)quinolin-4-amine (**JR3**) were purchased from Sigma-Aldrich (20 cm × 20 cm, 2 mm). NMR analyses were recorded on a Bruker Avance III 500 MHz. The corresponding ^1^H and ^13^C chemical shifts are reported relative to residual solvent protons and carbons.

### 3.2. Instrumentation

HPLC analyses were performed on a Merck-Hitachi L7000 (Hitachi, Ltd., Tokyo, Japan). High resolution ESI-MS was performed on a Bruker FTMS 4.7-T Apex II (Bruker Daltonics GmbH, Fällanden, Switzerland). NMR spectra were recorded on a Bruker Avance III 500 MHz (Bruker Daltonics GmbH, Fällanden, Switzerland). UV/Vis spectra were recorded on a Jasco V-730 (JASCO Deutschland GmbH, Pfungstadt, Germany) and the emission on a spectrofluorometer FS5 (Edinburgh Instruments Ltd., Livingston, UK). Titration experiments were performed as described by Egan et al. [49]. with the only exception that PBS (Phosphate-Buffered Saline) (1X, pH 7.4) was used instead of the *N*-[2-hydroxethyl]piperazine-*N*′-[2-ethane sulphonate] (HEPES) buffer. Crystallographic data were recorded on a SuperNova, Dual, Cu at zero, AtlasS2 diffractometer (Agilent Technologies XRD Products, Santa Clara, CA, USA). For biological studies, the extent of MTT reduction was measured spectrophotometrically via a Tecan Infinite 200 Pro multiplate reader (Tecan Group Ltd., Männedorf, Switzerland). Microscopy and fluorescent images were recorded with an inverted microscope (CKX41; Olympus, Tokyo, Japan) and a fluorescent microscope (Olympus BX51, Applied Imaging Corp., San Jose, CA, USA) respectively.

### 3.3. Computational Details 

Geometry optimizations as well as frequency calculations (gas phase), were performed at the Density Functional level of theory with the Gaussian09 program package (AMD64, Gaussian Inc., Wallingford, CT, USA) using the hybrid B3LYP [53] or M06 [54,55] functional in conjunction with the LanL2DZ basis set [56,57,58]. Pure basis functions (5d, 7f) were used in all calculations. Geometries were optimized without symmetry restrictions. The nature of the stationary points was checked by computing vibrational frequencies in order to verify true minima.

### 3.4. General Synthesis Procedures

Synthesis procedures for 7-chloro-*N*-(2-(4-(4-ethynylphenyl)-1*H*-1,2,3-triazol-1-yl)ethyl)quinolin-4-amine (compound **JR1**), a mixture of *N*-(2-azidoethyl)-7-chloroquinolin-4-amine (N_3_-CQ) (100 mg, 0.4 mmol, 1 eq.), 1,4-diethynylbenzene (60.5 mg, 0.5 mmol, 1.2 eq.) and tris(benzyltriazolylmethyl)amine (TBTA, 2 mg, 0,004 mmol, 1 mol-%) was dissolved in DMF (0.7 mL). To this solution was added a water mixture (0.3 mL) of CuSO_4_·5H_2_O (1 mg, 0,004 mmol, 1 mol-%) and sodium ascorbate (8 mg, 0,04 mmol, 0,1 eq.). The compounds were reacted overnight with stirring at room temperature. The compounds 7-chloro-*N*-(2-(4-(4-ethynyl-3-fluorophenyl)-1*H*-1,2,3-triazol-1-yl)ethyl)quinolin-4-amine (**JR2**) and 7-chloro-*N*-(2-(4-(4-ethynyl-2-fluorophenyl)-1*H*-1,2,3-triazol-1-yl)ethyl)quinolin-4-amine (**JR3**) were prepared similarly by replacing the 1,4-diethynylbenzene with 1,4-diethynyl-2-fluorobenzene (72 mg, 0.5 mmol, 1.2 eq.). Following the reaction, the solution containing **JR1** was filtered and purified by HPLC (system 2). The crude reaction containing both **JR2** and **JR3** compounds was also filtered and the filtrate freeze-dried. The remaining solid was redissolved in the minimal amount of DMF (0.2 mL) and completed with DCM/MeOH (0.4 mL, 9:1). The isomers were separated on a preparative TLC plate using DCM/MeOH (9:1) as eluent.

*7-Chloro-N-(2-(4-(4-ethynylphenyl)-1H-1,2,3-triazol-1-yl)ethyl)quinolin-4-amine* (**JR1**): yield 123 mg (0.033 mmol, 83%). ^1^H NMR (500 MHz, DMF-[d7]): δ = 9.68 (t, *J* = 5.6 Hz, 1H), 8.82 (s, 1H), 8.70 (d, *J* = 6.9 Hz, 1H), 8.60 (d, *J* = 9.0 Hz, 1H), 8.23 (d, *J* = 2.0 Hz, 1H), 8.03 (s, 1H), 7.89 (d, *J* = 8.45 Hz, 2H), 7.70 (dd, *J* = 7.76 Hz, 1H), 7.58 (dd, *J* = 8.35 Hz, 1H), 7.16 (d, *J* = 7Hz, 1H), 4.97 (t, *J* = 5.7 Hz, 2H), 4.31 (dd, *J* = 5.3, 5.8 Hz, 2H), 4.20 (s, 1H) ppm; ^13^C NMR (125 MHz, CDCl_3_-[d1]): δ= 165.4, 157.1, 146.4, 145.4, 141.1, 139.6, 135.8, 135.3, 128.4, 126.3, 124.5, 122.4, 121.6, 117.4, 113.0, 110.5, 100.1, 86.9, 77.8, 49.5, 44.5 ppm; HR-ESI-MS (ESI^+^): [M + H]^+^ = 374.1176, calculated for C_21_H_17_N_5_Cl_1_ = 374.1167.

*7-Chloro-N-(2-(4-(4-ethynyl-3-fluorophenyl)-1H-1,2,3-triazol-1-yl)ethyl)quinolin-4-amine* (**JR2**): yield 67 mg (0.017 mmol, 43%). ^1^H NMR (500 MHz, DMF-[d7]): δ = 9.59 (t, *J* = 5.86 Hz, 1H), 8.88 (s, 1H), 8.74 (d, *J* = 6.9 Hz, 1H), 8.56 (d, *J* = 9.0 Hz, 1H), 8.19 (d, *J* = 2.0 Hz, 1H), 7.79 (dd, *J* = 2.0, 9.0 Hz, 1H), 7.75 (t, *J* = 1.45 Hz, 1H), 7.73 (dd, *J* = 1.45, 4.5 Hz, 1H), 7.66 (t, *J* = 7.7 Hz, 1H), 7.16 (d, *J* = 6.8Hz, 1H), 4.98 (t, *J* = 6.15 Hz, 2H), 4.54 (s, 1H), 4.31 (dd, *J* = 5.5, 5.8 Hz, 2H) ppm; ^13^C NMR (125 MHz, CDCl_3_-[d1]): δ = 156.3, 146.5, 144.3, 140.0, 138.8, 132.8 (2C), 132.0, 127.5, 125.6 (3C), 123.0, 121.8, 120.4, 116.5, 99.3, 83.7, 80.6, 48.5, 43.8 ppm; HR-ESI-MS (ESI^+^): [M + H]^+^ = 392.1067, calculated for C_21_H_16_N_5_Cl_1_ = 392.1072.

*7-Chloro-N-(2-(4-(4-ethynyl-2-fluorophenyl)-1H-1,2,3-triazol-1-yl)ethyl)quinolin-4-amine* (**JR3**): yield 62 mg (0.16 mmol, 40%). ^1^H NMR (500 MHz, DMF-[d7]): δ = 9.68 (t, *J* = 5.96 Hz, 1H), 8.75 (d, *J* = 7.14 Hz, 1H), 8.68 (d, *J* = 3.8 Hz, 1H), 8.60 (d, *J* = 9.2 Hz, 1H), 8.21 (t, *J* = 8.0 Hz, 1H), 8.20 (d, *J* = 2.0, 1H), 7.81 (dd, *J* = 2.0, 9.14 Hz, 1H), 7.49 (dd, *J* = 1.55, 3.8 Hz, 1H), 7.48 (dd, *J* = 1.35, 7.05 Hz, 1H), 7.20 (d, *J* = 7.0 Hz, 1H), 5.02 (t, *J* = 5.8 Hz, 2H), 4.35 (s, 1H), 4.35 (dd, *J* = 5.5, 5.8 Hz, 2H) ppm; ^13^C NMR (125 MHz, CDCl_3_-[d1]): δ= 160.0, 158.5, 157.3, 144.9, 140.7, 140.5, 139.8, 129.8, 128.6, 126.4, 126.1, 124.1, 121.0, 120.3, 117.2, 100.2, 83.2, 82.7, 49.4, 44.6 ppm; HR-ESI-MS (ESI^+^): [M + H]^+^ = 392.1067, calculated for C_21_H_16_N_5_Cl_1_ = 392.1072

The synthesis of the derivatives cobalamine-1,4-diethynylbenzene (B_12-_**1**), cobalamine-1,4-diethynyl-1-fluorobenzene (B_12_-**F1**) and cobalamine-1,4-diethynyl-2-fluorobenzene (B_12_-**F2**) was adapted from the reference 29. A mixture of cyanocobalamin (60 mg, 0.04 mmol, 1 eq.), CuAcO (7 mg, 0.004 mmol, 0.1 eq.) and the respective alkynes (0.4 mmol, 10 eq.) in DMA (10 mL) was stirred until dissolution. DBU (0.03 mL, 0.21 mmol, 5 eq.) was added and the solutions allowed to react at room temperature for 4 h. The respective crudes were precipitated by dropwise addition to the stirred solutions of diethyl ether/CH_2_Cl_2_ (150 mL, 1:1). The residues were dissolved in a mixture of CH_3_OH and water (1.5 mL, 1:1), filtered again and purified by preparative HPLC using system 1 for the mixture B_12_-**F1** and B_12_-**F2** and system 2 for B_12-_**1**. The eluting bands containing the desired products were isolated and lyophilized. The antimalarial B_12_ were similarly prepared by Cu(I)-catalyzed azide-alkyne cycloaddition reaction. For the compound cobalamin-7-chloro-*N*-(2-(4-(4-ethynylphenyl)-1*H*-1,2,3-triazol-1-yl)ethyl)quinolin-4-amine (B_12_-**JR1**), a mixture of N_3_-CQ (10 mg, 0.04 mmol, 1 eq.) and TBTA (1 mg, 0,002 mmol, 5 mol-%) was dissolved in DMF (0.6 mL). To this solution was added a water mixture (0.4 mL) of B_12-_**1** (60 mg, 0.04 mmol, 1 eq.) CuSO_4_·5H_2_O (0.5 mg, 0,002 mmol, 5 mol-%) and sodium ascorbate (0.8 mg, 0,004 mmol, 0,1 eq.). The compounds were reacted overnight with stirring at room temperature. For the compounds cobalamin-7-chloro-*N*-(2-(4-(4-ethynyl-3-fluorophenyl)-1H-1,2,3-triazol-1-yl)ethyl)quinolin-4-amine (B_12_-**JR2**) and cobalamin-7-chloro-*N*-(2-(4-(4-ethynyl-2-fluorophenyl)-1*H*-1,2,3-triazol-1-yl)ethyl)quinolin-4-amine (B_12_-**JR3**), the same procedure was repeated but cyanocobalamin was replaced by the respective B_12_ precursors, B_12_-**F1** and B_12_-**F2** respectively (59 mg, 0.04 mmol, 1 eq.). The residues were dissolved in a mixture of CH_3_OH and water (1.5 mL, 1:1), filtered again and purified by preparative HPLC using system 2 for both B_12_-**JR2** and B_12_-**JR3**. The eluting bands containing the desired products were isolated and lyophilized.

*Cobalamine-1,4-diethynyl-1-fluorobenzene* (B_12_-**F1**): Yield 24.2 mg (0,017 mmol, 41%). ^1^H NMR (500 MHz, MeOD-[*d*4]): δ = 7.24 (s, 2H), 7.02 (s, 1H), 7.01 (d, *J* = 7.9 Hz, 1H), 6.81 (d, *J* = 7.9 Hz, 1H), 6.65 (s, 1H), 6.28 (d, *J* = 3.12 Hz, 1H), 6.02 (s, 1H), 4.73–4.65 (m, 1H), 4.63 (d, *J* = 8.25 Hz, 1H), 4.44 (d, *J* = 11.3 Hz, 1H), 4.39–4.31 (m, 1H), 4.22 (d, *J* = 3.6 Hz, 1H), 4.15–4.09 (m, 1H), 3.94 (dd, *J* = 3.2, 12.7 Hz, 1H), 3.78 (dd, *J* = 4.3, 12.7 Hz, 1H), 3.69 (d, *J* = 14.0 Hz, 1H), 3.64 (q, *J* = 5Hz, 1H), 3.56 (s, 1H), 3.24 (d, *J* = 10.0 Hz, 1H), 2.92–2.82 (m, 2H), 2.65–2.59 (m, 16H), 2.57 (d, *J* = 4.88 Hz, 6H), 2.56–2.50 (m, 4H), 2.35 (s, 1H), 2.30 (d, *J* = 3 Hz, 3H), 2.28–2.19 (m, 2H), 2.13–1.91 (m, 6H), 1.87 (s, 3H), 1.86–1.70 (m, 3H), 1.48 (s, 3H), 1.41–1.38 (m, 1H), 1.37 (m, 3H), 1.35–1.32 (m, 1H), 1.27 (d, *J* = 6.24 Hz, 3H), 1.24–1.16 (m, 1H), 1.14 (s, 3H), 0.52 (s, 3H) ppm; HR-ESI-MS (ESI^+^): [M + H]^+^ = 1472.5989, calculated for C_72_H_93_N_13_O_14_Co_1_P_1_F_1_ = 1472.6013

*Cobalamine-1,4-diethynyl-2-fluorobenzene* (B_12_-**F2**): Yield 22.5 mg (0,015 mmol, 38%). ^1^H NMR (500 MHz, MeOD-[d4]): δ = 7.24 (d, *J* = 4.32 Hz, 1H), 7.20 (t, *J* = 7.82 Hz, 1h), 6.64 (ps, 1H), 6.62 (d, *J* = 7.17 Hz, 1H), 6.56 (d, *J* = 10.4 Hz, 1H), 6.28 (d, *J* = 3.12 Hz, 1H), 6.02 (s, 1H), 4.73–4.65 (m, 1H), 4.63 (d, *J* = 8.25 Hz, 1H), 4.44 (d, *J* = 11.3 Hz, 1H), 4.39–4.31 (m, 1H), 4.22 (d, *J* = 3.6 Hz, 1H), 4.15–4.09 (m, 1H), 3.94 (dd, *J* = 3.2, 12.7 Hz, 1H), 3.78 (dd, *J* = 4.3, 12.7 Hz, 1H), 3.69 (d, *J* = 14.0 Hz, 1H), 3.64 (q, *J* = 5Hz, 1H), 3.56 (s, 1H), 3.24 (d, *J* = 10.0 Hz, 1H), 2.92–2.82 (m, 2H), 2.65–2.59 (m, 16H), 2.57 (d, *J* = 4.88 Hz, 6H), 2.56–2.50 (m, 4H), 2.35 (s, 1H), 2.30 (d, *J* = 3 Hz, 3H), 2.28–2.19 (m, 2H), 2.13–1.91 (m, 6H), 1.87 (s, 3H), 1.86–1.70 (m, 3H), 1.48 (s, 3H), 1.41–1.38 (m, 1H), 1.37 (m, 3H), 1.35–1.32 (m, 1H), 1.27 (d, *J* = 6.24 Hz, 3H), 1.24–1.16 (m, 1H), 1.14 (s, 3H), 0.52 (s, 3H) ppm; HR-ESI-MS (ESI^+^): [M + H]^+^ = 1472.5989, calculated for C_72_H_93_N_13_O_14_Co_1_P_1_F_1_ = 1472.6013

*Cobalamin-7-chloro-N-(2-(4-(4-ethynylphenyl)-1H-1,2,3-triazol-1-yl)ethyl)quinolin-4-amine* (B_12_-**JR1**): Yield 51.6 mg (0,03 mmol, 75%). ^1^H NMR (500 MHz, D_2_O-[d1]): δ = 8.10 (s, 1H), 8.08 (d, *J* = 7.30 Hz, 1H), 7.95 (d, *J* = 9.1 Hz, 1H), 7.49 (d, *J* = 1.9 Hz, 1H), 7.39 (dd, *J* = 1.9, 9.13 Hz, 1H), 7.26 (d, *J* = 1.45 Hz, 2H), 7.23 (s, 1H), 7.10 (s, 1H), 6.89 (d, *J* = 8.3 Hz, 2H), 6.54 (s, 1H), 6.48 (d, *J* = 7.25 Hz, 1H), 6.35 (d, *J* = 2.91 Hz, 1H), 5.94 (s, 1H), 4.76–4.70 (m, 2H), 4.36–4.24 (m, 4H), 4.18–4.12 (m, 2H), 4.11–4.04 (m, 2H), 4.94 (d, *J* = 12.9 Hz, 1H), 3.77 (dd, *J* = 3.9, 12.9 Hz, 1H), 3.59 (d, *J* = 14.7 Hz, 1H), 3.20 (dd, *J* = 5.0, 11.0 Hz, 1H), 3.04 (d, *J* = 10.0 Hz, 1H), 2.96 (dd, *J* = 9.13, 14.3 Hz, 1H), 2.76–2.45 (m, 13H), 2.46–2.32 (m, 6H), 2.26 (s, 6H), 2.20–2.07 (m, 3H), 2.06–1.90 (m, 5H), 1.86 (s, 3H), 1.82–1.69 (m, 3H), 1.38 (s, 3H), 1.36 (s, 3H), 1.26 (d, *J* = 6.3 Hz, 3H), 1.18 (s, 3H), 1.13–1.01 (m, 2H), 0.97 (s, 3H), 0.46 (s, 3H) ppm; HR-ESI-MS (ESI^+^): [M + 2H]^2+^ = 851.3401, calculated for C_83_H_105_N_18_O_14_Co_1_P_1_Cl_1_ (2+) = 851.3402.

*Cobalamin-7-chloro-N-(2-(4-(4-ethynyl-3-fluorophenyl)-1H-1,2,3-triazol-1-yl)ethyl)quinolin-4-amine* (B_12_-**JR2**): Yield 41.7 mg (0,024 mmol, 60%). ^1^H NMR (500 MHz, D_2_O-[d1]): δ = 8.10 (s, 1H), 8.09 (d, *J* = 6.0 Hz, 1H), 8.01 (d, *J* = 8.0 Hz, 1H), 7.54 (d, *J* = 1.94 Hz, 1H), 7.48 (dd, *J* = 1.9, 9.3 Hz, 1H), 7.28 (s, 1H), 7.14 (s, 1H), 7.01 (s, 1H), 6.95 (d, *J* = 3.0 Hz, 1H), 6.90 (d, *J* = 7.8 Hz, 1H), 6.56 (s, 1H), 6.48 (d, *J* = 7.3 Hz, 1H), 6.37 (d, *J* = 3.13 Hz, 1H), 6.0 (s, 1H), 4.76–4.70 (m, 2H), 4.36–4.24 (m, 4H), 4.18–4.12 (m, 2H), 4.11–4.04 (m, 2H), 4.94 (d, *J* = 12.9 Hz, 1H), 3.77 (dd, *J* = 3.9, 12.9 Hz, 1H), 3.59 (d, *J* = 14.7 Hz, 1H), 3.20 (dd, *J* = 5.0, 11.0 Hz, 1H), 3.04 (d, *J* = 10.0 Hz, 1H), 2.96 (dd, *J* = 9.13, 14.3 Hz, 1H), 2.76–2.45 (m, 13H), 2.46–2.32 (m, 6H), 2.26 (s, 6H), 2.20–2.07 (m, 3H), 2.06–1.90 (m, 5H), 1.86 (s, 3H), 1.82–1.69 (m, 3H), 1.38 (s, 3H), 1.36 (s, 3H), 1.26 (d, *J* = 6.3 Hz, 3H), 1.18 (s, 3H), 1.13–1.01 (m, 2H), 0.97 (s, 3H), 0.38 (s, 3H) ppm; HR-ESI-MS (ESI^+^): [M + Na]^+^ = 1741.6457, calculated for C_83_H_102_ N_18_O_14_Co_1_P_1_F_1_Na_1_ = 1741.6457.

*Cobalamin-7-chloro-N-(2-(4-(4-ethynyl-2-fluorophenyl)-1H-1,2,3-triazol-1-yl)ethyl)quinolin-4-amine* (B_12_-**JR3**): Yield 48.8 mg (0.028 mmol, 75%). ^1^H NMR (500 MHz, D_2_O-[d1]): δ = 8.00 (d, *J* = 7.0 H 1H), 7.97 (s, 1H), 7.46 (t, *J* = 7.9 Hz, 1H), 7.31 (ps, 1H), 7.17 (s, 1H), 7.09 (d, *J* = 8.6 Hz, 1H), 7.00 (s, 1H), 6.63 (d, *J* = 8.09 Hz, 1H), 6.45 (d, *J* = 9.6 Hz, 1H), 6.43 (s, 1H), 6.38 (d, *J* = 6.8 Hz, 1H), 6.26 (d, *J* = 2.8 Hz, 1H), 5.8 (s, 1H), 4.76–4.70 (m, 2H), 4.36–4.24 (m, 4H), 4.18–4.12 (m, 2H), 4.11–4.04 (m, 2H), 4.94 (d, *J* = 12.9 Hz, 1H), 3.77 (dd, *J* = 3.9, 12.9 Hz, 1H), 3.59 (d, *J* = 14.7 Hz, 1H), 3.20 (dd, *J* = 5.0, 11.0 Hz, 1H), 3.04 (d, *J* = 10.0 Hz, 1H), 2.96 (dd, *J* = 9.13, 14.3 Hz, 1H), 2.76–2.45 (m, 13H), 2.46–2.32 (m, 6H), 2.26 (s, 6H), 2.20–2.07 (m, 3H), 2.06–1.90 (m, 5H), 1.86 (s, 3H), 1.82–1.69 (m, 3H), 1.38 (s, 3H), 1.28 (s, 3H), 1.24 (s, 3H), 1.17 (d, *J* = 6.23 Hz, 3H), 1.03 (s, 1H), 0.78 (s, 3H), 0.36 (s, 3H) ppm; HR-ESI-MS (ESI^+^): [M + Na]^+^ = 1741.6457, calculated for C_83_H_102_ N_18_O_14_Co_1_P_1_F_1_Na_1_ = 1741.6457.

Synthesis of 2-azido-1-(2-(4-fluorophenyl)-3-((4-fluorophenyl)amino)-8,8-dimethyl-5,6-dihydroimidazo[1,2-a]pyrazin-7(8*H*)-yl)ethan-1-one **(N_3_-SN1)**: To the solution of KAF156 (100 mg, 0.024 mmol, 1.0 eq) in dry acetonitrile (10 mL) under argon, 2-azido-1,3-dimethylimidazolinium hexafluorophosphate (90 mg, 0.031 mmol, 1.3 eq) and trimethylamine andydruos (180 μL,0.121 mmol, 5.0 eq) were added. The mixture was then heated to 30 °C and stirred for 4 h. After the reaction completion, the solvent was evaporated, DCM (20 mL) and water (20 mL) were added to the residue and the two phases were separated. The aqueous phase was extracted with 2 × 20 mL of CH_2_Cl_2_. The combined organic layers were washed with brine (20 mL), separated and dried over Na_2_SO_4_. The mixture was filtered and the solvent was removed under reduced pressure. The crude product was purified by flash column chromatography to afford compound N_3_-**SN1** as a white solid. Yield: 85 mg (0.194 mmol, 80%). ^1^H NMR (400 MHz, MeOD-[d4]): δ = 7.73 (dd, *J* = 5.2, 5.92 Hz, 2H), 7.04 (dd, *J* = 8.68 Hz, 2H), 6.91 (dd, *J* = 8.96 Hz, 2H), 6.57 (dd, *J* = 4.32, 6.8 Hz, 2H), 4.17 (s, 2H), 3.84 (t, *J* = 5.36, 2H), 3.68 (t, *J* = 5.76, 2H), 1.97 (s, 6H), = 5.8 HR-ESI-MS (ESI^+^): [M + H]^+^ = 437.47, calculated for C_22_H_21_F_2_N_7_O = 437.17.

Synthesis of 2-(4-(4-ethynylphenyl)-1H-1,2,3-triazol-1-yl)-1-(2-(4-fluorophenyl)-3-((4-fluorophenyl)amino)-8,8-dimethyl-5,6-dihydroimidazo[1,2-a]pyrazin-7(8*H*)-yl)ethan-1-one (**SN1**): A mixture of N_3_-**SN1** (85 mg, 0.194 mmol, 1 eq.), 1,4-diethynylbenzene (29.40 mg, 0.233 mmol, 1.2 eq.) and Et_3_N (7.87 mg, 0.077 mmol, 40 mol-%) was dissolved in DMF (1 mL). CuI (3.70 mg, 0.019 mmol, 10 mol-%) was added to this solution. The compounds were reacted overnight with stirring at room temperature. After the reaction completion, brine (20 mL) and dichloromatane (20 mL) were added to the solution. The aqueous phase was extracted with 2 × 20 mL of CH_2_Cl_2_. The combined organic layers were washed with brine (20 mL) and the organic layer separated and dried over Na_2_SO_4_. Then, the mixture was filtered and finally CH_2_Cl_2_ was removed under reduced pressure. The crude product was purified by flash column chromatography to afford compound **SN1** as a white solid. Yield: 93 mg (0.165 mmol, 85%). ^1^H NMR (400 MHz, (CD_3_)_2_CO-[d6]): δ = 8.22 (s, 2H), 7.78 (m, 4H), 7.43 (d, *J* = 8.4Hz, 2H), 6.92 (d, *J* = 8.6 2H), 6.82 (dd, *J* = 8.56 2H), 6.56 (dd, *J* = 4.48, 8.92 Hz 2H), 5.52 (s, 2H), 3.89 (t, *J* = 5.6 Hz, 2H), 3.85 (t, *J* = 5.40 Hz, 2H), 3.55 (s, 1H), 1.77 (s, 6H); HR-ESI-MS (ESI^+^): [M + H]^+^ = 564.23, calculated for C_32_H_28_F_2_N_7_O = 564.23.

Synthesis of Cobalamin-2-(4-(4-ethynylphenyl)-1*H*-1,2,3-triazol-1-yl)-1-(2-(4-fluorophenyl)-3-((4-fluorophenyl)amino)-8,8-dimethyl-5,6-dihydroimidazo[1,2-a]pyrazin-7(8*H*)-yl)ethan-1-one (**B_12_-SN1**): a mixture of N_3_-**SN1** (50 mg, 0.114 mmol, 1 eq.) and TBTA (3.03 mg, 0,005 mmol, 5 mol-%) was dissolved in DMF (1 mL). To this solution a water mixture (0.4 mL) of B_12-_**1** (166 mg, 0.114 mmol, 1 eq.) CuSO_4_·5H_2_O (1.42 mg, 0,005 mmol, 5 mol-%) and sodium ascorbate (2.26 mg, 0,011 mmol, 0.1 eq.) was added. The mixture stirred overnight at room temperature. The residues were dissolved in a mixture of CH_3_OH and water (1.5 mL, 1:1), filtered again and purified by preparative HPLC using. The eluting bands containing the desired products were isolated and lyophilized. The pure product (B_12_-**SN1**) was obtained as a red solid. Yield: 116.70 mg (0.080 mmol, 70%); ^1^H NMR (400 MHz, D_2_O-[d1]): δ = 8.19 (s, 1H), 7.73 (dd, *J* = 5.4, 14.4 Hz, 2H), 7.57 (d, *J* = 8.3 Hz, 1H), 7.25 (s, 2H), 7.04 (t, *J* = 6.84 Hz, 1H), 6.92 (dd, *J* = 8.4 14.96 Hz, 4H), 6.66 (s, 1H), 6.58 (dd, *J* = 4.4, 4.52 2H), 6.23 (d, *J* = 3.12 Hz, 1H), 6.02 (s, 1H), 5.56 (d, *J* = 7.0 Hz, 1H), 4.76–4.60 (m, 3H), 4.45 (d, *J* = 11.48, 1H), 4.4–4.30 (m, 1H), 4.22 (t, *J* = 3.6, 1H), 4.16–4.10 (m, 1H), 3.95 (dd, *J* = 1.62, 11.02 Hz, 1H), 3.79 (dd, *J* = 4.36, 10.42 Hz, 1H), 3.69 (d, *J* = 13.88 Hz, 1H), 3.59 (dd, *J* = 4.96, 8.16 Hz, 1H), 3.26 (d, *J* = 9.76 Hz, 1H), 2.87 (m, 2H), 2.66–2.2 (m, 13H), 2.50–2.43 (m, 3H), 2.37 (d, *J* = 13.2, 1H), 2.3 (d, *J* = 4.6, 6H), 2.27–2.21 (m, 1H), 1.95 (s, 6H), 1.89 (s, 3H), 1.85–1.71 (m, 2H), 1.49 (S, 3H), 1.38 (s, 3H), 1.34 (s, 3H), 1.27 (d, *J* = 6.3 Hz, 3H), 1.17 (s, 3H), 0.53 (s, 3H) ppm; HR-ESI-MS (ESI^+^): [M + 2H]^2+^ = 945.3402, calculated for [C_95_H_115_CoF_2_N_19_O_15_P]^2+^ = 945.9.

### 3.5. In Vitro Cytotoxicity Assay 

B_12_-**JR1** was screened for in vitro cytotoxicity against the Chinese Hamster Ovarian (CHO) cell-line using the 3-(4,5-dimethylthiazol-2-yl)-2,5-diphenyltetrazolium bromide (MTT)-assay [59]. The test sample was prepared to a 20 mg mL^−1^ stock solution in 100% DMSO and tested in triplicate. Stock solutions were stored at −20 °C. Further dilutions were prepared in complete medium on the day of the experiment. Samples were tested as a suspension if not completely dissolved. Emetine was used as the reference drug in all experiments. The initial concentration of emetine was 100 µg mL^−1^, which was diluted in complete medium with 10-fold dilutions to give 6 concentrations, the lowest being 0.001 µg mL^−1^. The same dilution technique was applied to all test samples. The highest concentration of solvent to which the cells were exposed to have no measurable effect on the cell viability. The 50% inhibitory concentration (IC_50_) values were obtained from full dose-response curves, using a non-linear dose-response curve fitting analysis via GraphPad Prism v.5 software. Furthermore, the cytotoxicity in terms of the antiproliferative effect of B_12_-**JR1**, B_12_-**JR2** and B_12_-**JR3** were similarly evaluated by the MTT assay. The assay was carried out using human lung fibroblasts (MRC5) cell line after 48 h of cell incubation in the medium, containing compounds at concentrations ranging from 5 to 200 μM. Briefly, MRC5 cells were maintained in RPMI-1640 medium supplemented with 100 μg/mL streptomycin, 100 U/mL penicillin and 10% (v/v) fetal bovine serum (FBS) (Gibco) as a monolayer (1 × 10^4^ cells per well). All cell lines were grown in humidified atmosphere of 95% air and 5% CO_2_ at 37 °C. The MTT assay was performed two times in four replicates. The extent of MTT reduction was measured spectrophotometrically at 540 nm using a Tecan Infinite 200 Pro multiplate reader (Tecan Group Ltd., Männedorf, Switzerland), and the cell survival was expressed as percentage of the control (untreated cells). Cytotoxicity was expressed as the concentration of the compound inhibiting cell growth by 50% (IC_50_) in comparison to untreated control. The results were expressed as mean values ± standard deviation (SD) and analysed using Student’s t-test at a threshold level of *p* = 0.05. Statistical analysis was carried out using SPSS 20 (SPSS Inc., Chicago, IL, USA) software. All experiments were conducted in at least three replicates.

### 3.6. In Vitro Antiplasmodial Assay 

The test samples were tested in triplicate against the chloroquine-sensitive (CQS) strain of *Plasmodium falciparum* (NF54) and the CQ-resistant isolate (K1). Continuous in vitro cultures of asexual erythrocyte stages of *P. falciparum* were maintained using a modified method by Trager and Jensen [60]. Quantitative assessment of the antiplasmodial activity in vitro was determined via the parasite lactate dehydrogenase assay using a modified method described by Makler [61]. The test samples were prepared to a 20 mg/mL stock solution in 100% DMSO and sonicated to enhance solubility. Samples were tested as a suspension if not completely dissolved. Stock solutions were stored at −20 °C. Further dilutions were prepared on the day of the experiment. Chloroquine (CQ) was used as the reference drug in all experiments. A full dose-response was performed for all compounds to determine the concentration inhibiting 50% of parasite growth (IC_50_ value). The vitamin B_12_ complexes were tested at starting concentrations up to 50 μg/ mL, which was then serially diluted 2-fold in complete medium to give 10 concentrations. The ligands were tested at a starting concentration of 10 μg/mL, which was serially diluted 2-fold in complete medium to give 10 concentrations. CQ was tested at a starting concentration of 1 μg/mL. The IC_50_ values were obtained using a non-linear dose-response curve fitting analysis via Graph Pad Prism v.5.0 software (GraphPad Software Inc., San Diego, CA, USA).

### 3.7. In Vitro Blood Assays 

B_12_-**JR1**-**CBC** was used to study in vitro bio-distribution in blood using both whole-blood and washed red blood cells. Blood was collected from a healthy dog with the consent of owner into BD Vacutainer^®^ Plus plastic citrate tube (2 mL; haematocrit value of 40, Vetlab Animal Diagnostic Laboratory, Belgrade, Serbia; http://www.vetlab.rs/) and used immediately as a whole-blood sample and for the red blood cells (RBC) preparation. The platelet rich plasma and buffy coat were discarded after centrifugation (370× *g*, 10 min). The erythrocytes were then washed in 10 times their volume of phosphate-buffered saline (PBS) and resuspended in isotonic PBS. The haemoglobin concentration was adjusted to 2 g/100 mL. To the whole-blood and to the RBC suspension B_12_-**JR1**-**CBC** (20 µM final concentration) was added. The final incubation volume was 1 mL and the tubes were shaken on rocking platform at 37 °C for 24 h in dark. At different incubation periods (30 min, 2 h, 8 h, 12 h and 24 h) 5 µL of the whole sample was spread on the microscopic slide for the direct examination (fluorescence microscope Olympus BX51, Applied Imaging Corp., San Jose, CA, United States; Texas Red filter), while 100 µL of RBC incubation mixture was transferred into 0.5 mL Eppendorf tube, centrifuged briefly (370× *g*, 1 min) and 50 µL sample of the supernatant diluted 10-fold in PBS and analysed for B12-JR1-CBC presence using Tecan Infinite 200 Pro multiplate reader (Tecan Group, Männedorf, Switzerland; λEx = 488 nm, λEm = 540 nm). The assay was performed three times in duplicate and the results were presented as percentage of the control (RBC suspension the time of the compound addition) that was arbitrarily set to 100%.

### 3.8. In Vivo Zebrafish Assays Toxicity

The toxicity evaluation of B_12_-**JR1**, B_12_-**JR1-CBC** and vitamin B_12_ in the zebrafish model was carried out according to general rules of the OECD Guidelines for the Testing of Chemicals [62]. All experiments involving zebrafish were performed in compliance with the European directive 2010/63/EU and the ethical guidelines of the Guide for Care and Use of Laboratory Animals of the Institute of Molecular Genetics and Genetic Engineering, University of Belgrade. Embryos of wild type zebrafish (*Danio rerio*, AB strain) were raised in a temperature- and light-controlled zebrafish facility with 28 °C and standard 14:10-h light-dark photoperiod, and regularly fed with commercially dry flake food (TetraMin™ flakes; Tetra Melle, Germany) twice a day and *Artemia nauplii* once daily. Zebrafish embryos were produced by the pair-wise mating, collected and distributed into 24-well plates containing 10 embryos per well and 1 mL embryos water (0.2 g L^−1^ of Instant Ocean^®^ Salt in distilled water), and raised at 28 °C. For assessing lethal and developmental toxicity, embryos staged 6 h post fertilization (hpf) were treated with four concentrations of B_12_-**JR1** and vitamin B_12_ (50, 75, 100 and 150 µM) and four concentrations of B_12_-**JR1-CBC** (20, 25, 50 and 100 µM). DMSO (0.25% *v*/*v*) was used as negative control. Experiments were performed three times using 30 embryos per concentration. Apical endpoints for the toxicity evaluation (Table S1) were recorded at 24, 48, 72, 96 and 120 hpf using an inverted microscope (CKX41; Olympus, Tokyo, Japan). Dead embryos were counted and discarded every 24 h. At 120 hpf, embryos were inspected for heartbeat rate, anesthetized by addition of 0.1% (*w*/*v*) tricaine solution (Sigma-Aldrich, St. Louis, MO, USA), photographed and killed by freezing at −20 °C for ≥24 h. To analyse compound B_12_-**JR1** and B_12_-**JR1-CBC** for possible hepatotoxic effect in vivo, the transgenic *Tg*(*fabp10*:EGFP) zebrafish embryos with the fluorescently labelled liver were exposed to the tested compounds at the 72 hpf stage, when the liver is fully functional, vascularized and started metabolic transformation of absorbed compounds, assessing thus the effect of applied compounds on the liver functioning. Embryos were exposed to the range of concentrations previously determined that not affected embryos survival and development. Experiments were performed three times using 5 embryos per concentration. The hepatotoxicity was determined according to the change of liver area index compared to the control group, calculated as the ration between liver area and embryonic lateral area x 100%, as reported by Zhang et al. [63]. In addition, the liver colour and retention of yolk were followed as the phenotypic signs of hepatotoxicity [64].

### 3.9. Distribution 

To determine the distribution and the accumulation of B_12_-**JR1** and B_12_-**JR1-CBC** through inner organs, transgenic *Tg*(*fabp*:EGFP) zebrafish embryos were exposed to a non-toxic concentration of each of the tested compounds (150 µM of B_12_-**JR1** and 20 µM B_12_-**JR1-CBC**) in a period from 72 to 120 hpf (a long exposure started when the liver became vascularized and capable to metabolize the absorbed compounds) and 106 to 120 hpf (a shorter exposure time). To verify a preferential accumulation of B_12_-**JR1-CBC** in the liver, the compound was applied intravenously (parenteral use) to zebrafish embryos. The 106-hpf old embryos were anesthetized by addition of tricaine-methane sulfonate (200 μg/mL, Sigma-Aldrich), and microinjected by a pneumatic picopump (PV820, World Precision Instruments, USA) with 5 nL of B_12_-**JR1-CBC** (9.56 µg/nL per an embryo, corresponding to 20 µM dose applied into embryo water). The treated embryos were analysed at 120 hpf by a fluorescent microscope (Olympus BX51, Applied Imaging Corp., San Jose, CA, USA) upon Texas Red filter (an excitation max 596 nm, an emission max 620 nm) and Spectrum Green filter (an excitation max 497 nm, an emission max 524 nm) to detect B_12_-**JR1-CBC** and the EGFP-labelled liver, respectively.

## 4. Conclusions

In summary, we have reported the synthesis of vitamin B_12_ derivatives designed for the delivery the same antimalarial prodrug to both erythro- and hepatocytes. The drugs are released from the Cbl scaffold with a 4-(4-ethynylphenyl)-triazole functionality. In this proof-of-concept study, the chloroquine molecules we designed were equally active against chloroquine-resistant (CQR) and chloroquine-sensitive (CQS) *P. falciparum* strains, and showed no toxicity in vitro and in vivo (zebrafish model) no hepatotoxicity, no cardiotoxicity or developmental toxicity of the embryos. While we are aware that this approach may not be cost effective for the treatment of a large number of individuals, it may lead to development of antimalarial drugs of last resort or new organic drugs candidates based on the triazole functionality we selected for this study.

## Figures and Tables

**Table 1:** Antiplasmodial activity of molecules against the chloroquine-sensitive (CQS) NF54 and chloroquine-resistant (CQR) K1 strains of *Plasmodium falciparum*.

Compound	NF54 IC_50_ ± SD (µM)	K1 IC_50_ ± SD (µM)	Resistance Index (RI) ^a^
**JR1**	0.14 ± 0.01	0.18 ± 0.02	1.3
**JR2**	0.15 ± 0.01	0.16 ± 0.01	1.1
**JR3**	0.19 ± 0.02	0.21 ± 0.02	1.1
B_12_-**JR1**	6.27 ± 0.37	9.18 ± 0.85	1.5
B_12_-**JR2**	4.73 ± 0.32	6.02 ± 0.71	1.3
B_12_-**JR3**	9.68 ± 0.73	>15	>1.6
**SN1**	na ^b^	nd ^c^	nd
B_12_-**SN1**	na	nd	nd
CQ	0.013 ± 0.001	0.40 ± 0.04	30.8

^a^ RI (resistance index): IC_50_ (CQR)/IC_50_ (CQS); ^b^ Not active at the tested concentration; ^c^ Not determined.

**Table 2:** Therapeutic windows of B_12_-**JR1** for NF54 strain (CQ-sensitive) and K1 strain (CQ-resistant) of *P. falciparum*.

	IC_50_ (µM) ^a^		LC_50_ (µM) ^b^	Ti (LC_50_/IC_50_)	
	NF54	K1		NF54	K1
B_12_-JR1	6.3	9.2	>150	>23.9	>16.3

^a^—in vitro data obtained in the cell-based assay; ^b^—in vivo data obtained in the zebrafish model.

## Supplementary Materials

The following are available online. Figure S1: 500 MHz ^1^H-NMR of compound JR1 (in DMF, * = solvent residual peak), Figure S2: 500 MHz ^1^H-NMR of compound JR2 (in DMF, * = solvent residual peak), Figure S3: 500 MHz ^1^H-NMR of compound JR3 (in DMF, * = solvent residual peak), Figure S4: 500 MHz ^1^H-NMR of compound B12-F1 (in MeOD, * = solvent residual peak), Figure S5: 500 MHz ^1^H-NMR of compound B12-F2 (in MeOD, * = solvent residual peak), Figure S6: 500 MHz ^1^H-NMR of compound B12-JR1 (in D_2_O, * = solvent residual peak), Figure S7: 500 MHz ^1^H-NMR of compound B12-JR2 (in D_2_O, * = solvent residual peak), Figure S8: 500 MHz ^1^H-NMR of compound B12-JR3 (in D_2_O, * = solvent residual peak), Figure S9: 400 MHz ^1^H-NMR of compound N3-SN1 (in MeOD, * = solvent residual peak), Figure S10: 400 MHz ^1^H-NMR of compound SN1 (in (CD_3_)_2_CO, * = solvent residual peak), Figure S11: 400 MHz ^1^H-NMR of compound B12-SN1 (in D_2_O, * = solvent residual peak), Figure S12: DFT optimized structure of *N*-quinoline bound JR1 model with ferriprotoporphyrin IX, Figure S13: DFT optimized structure of *N*-triazole bound JR1 model with ferriprotoporphyrin IX, Figure S14: DFT optimized structure of *N*-quinoline protonated JR1 model interacting with ferriprotoporphyrin IX μ-oxo dimer, Figure S15: HR-ESI-MS spectrum (in MeOH) of compound JR1, Figure S16: HR-ESI-MS spectrum (in MeOH) of compound JR2/3, Figure S17: HR-ESI-MS spectrum (in MeOH) of compound B12-F1/2, Figure S18: HR-ESI-MS spectrum (in MeOH) of compound B12-JR1, Figure S19: HR-ESI-MS spectrum (in MeOH) of compound B12-JR2/3, Figure S20: HR-ESI-MS spectrum (in MeOH) of compound B12-JR1-CBC, Figure S21: Top: distribution of compound B12-JR1-CBC in suspension of washed red blood cells (RBC) over time. RBC suspensions with (Sample) and without (Control) the molecule were incubated with 20 µM (final concentration) of B12-JR1-CBC at 37 °C for 24 h in dark with shaking and at various time points aliquots were centrifuged and the amount of fluorescence in the supernatant determined (λEx = 488 nm, λEm = 540 nm). Bottom: fluorescence spectra of full smear blood control, Figure S22: Calculated IR spectrum of DFT optimized structure (gas-phase) of the interaction of a protonated 4-(4-ethynylphenyl)-triazole functionalized quinoline drug model with ferriprotoporphyrin IX μ-oxo dimer shown in Figure 5 of the manuscript. Note no negative frequencies verifying a true minimum, Figure S23: Bio-distribution and accumulation of B12-JR1 and B12-JR1-CBC in the 120-hpf old transgenic Tg(fabp10:EGFP) zebrafish embryo with fluorescently labelled liver applied at different developmental stages, Figure S24: HPLC traces of molecules prepared in this study, Table S1: Crystallographic details of compound JR1, Table S2: Crystallographic details of compound B12-F2.
