# Supplementary material for: Antiplasmodial Activity and In Vivo Bio-Distribution of Chloroquine Molecules Released with a 4-(4-Ethynylphenyl)-Triazole Moiety from Organometallo-Cobalamins
Source: Molecules. 2019 Jun 21;24(12):2310. doi: 10.3390/molecules24122310 (PMC6630517; doi:10.3390/molecules24122310)
Supplement: Supplementary file 1 [file molecules-24-02310-s001.pdf]

Supporting Information F

Antiplasmodial Activity and In Vivo Bio-Distribution of Chloroquine Molecules Released with a 4-(4-Ethynylphenyl)-Triazole Moiety from Organometallo-Cobalamins

Jeremie Rossier [1,†], Sara Nasiri Sovari [1,†], Aleksandar Pavic [2,†], Sandra Vojnovic [2], Tameryn Stringer [3], Sarah Bättig [1], Gregory S. Smith [3], Jasmina Nikodinovic-Runic [2,*] and Fabio Zobi [1,*]

[1] Department of Chemistry, University of Fribourg, Chemin du Musée 10, 1700 Fribourg, Switzerland; jeremie.rossier@unifr.ch (J.R.); sara.nasirisovari@unifr.ch (S.N.S.); sarah.baettig@unifr.ch (S.B.)
[2] Institute of Molecular Genetics and Genetic Engineering, University of Belgrade, Vojvode Stepe 444a, 11000 Belgrade, Republic of Serbia; pavicaleksandarr@gmail.com (A.P.); sanvojnov@gmail.com (S.V.)
[3] Department of Chemistry, University of Cape Town, Rondebosch 7701, South Africa; STRTAM001@myuct.ac.za (T.S.); gregory.smith@uct.ac.za (G.S.S.)
***** Correspondence: jasmina.nikodinovic@imgge.bg.ac.rs (J.N.-R.); fabio.zobi@unifr.ch (F.Z.); Tel: +381-113976034 (J.N.-R.); +41-26-300-8785 (F.Z.)
[†] These authors contributed equally to this work.

NMR spectra

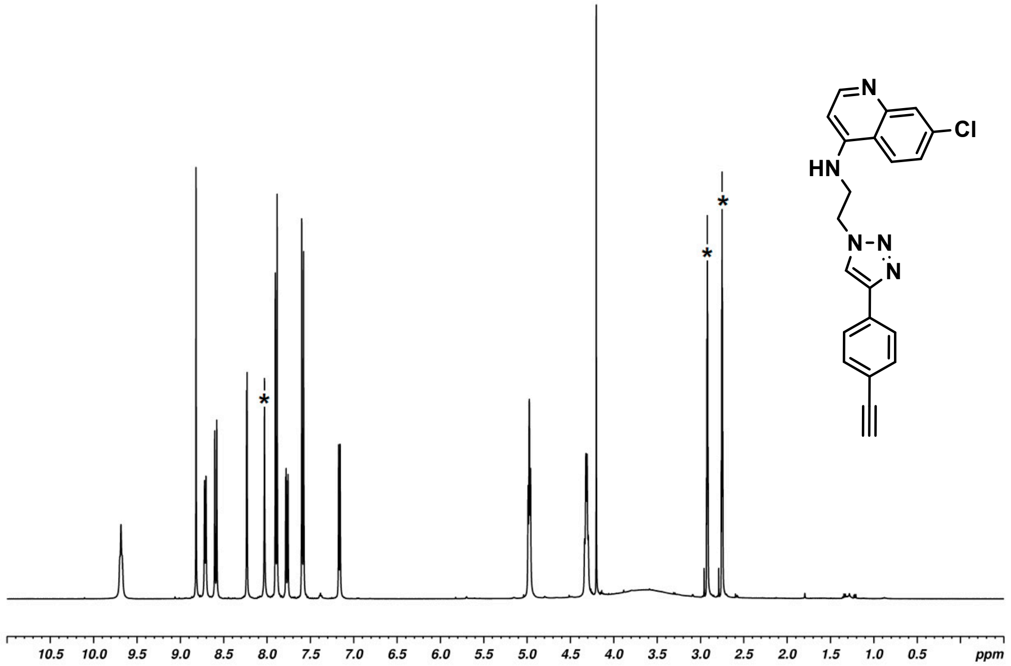

Figure S1. 500 MHz ^{1}H-NMR of compound **JR1** (in dDMF, * = solvent residual peak)

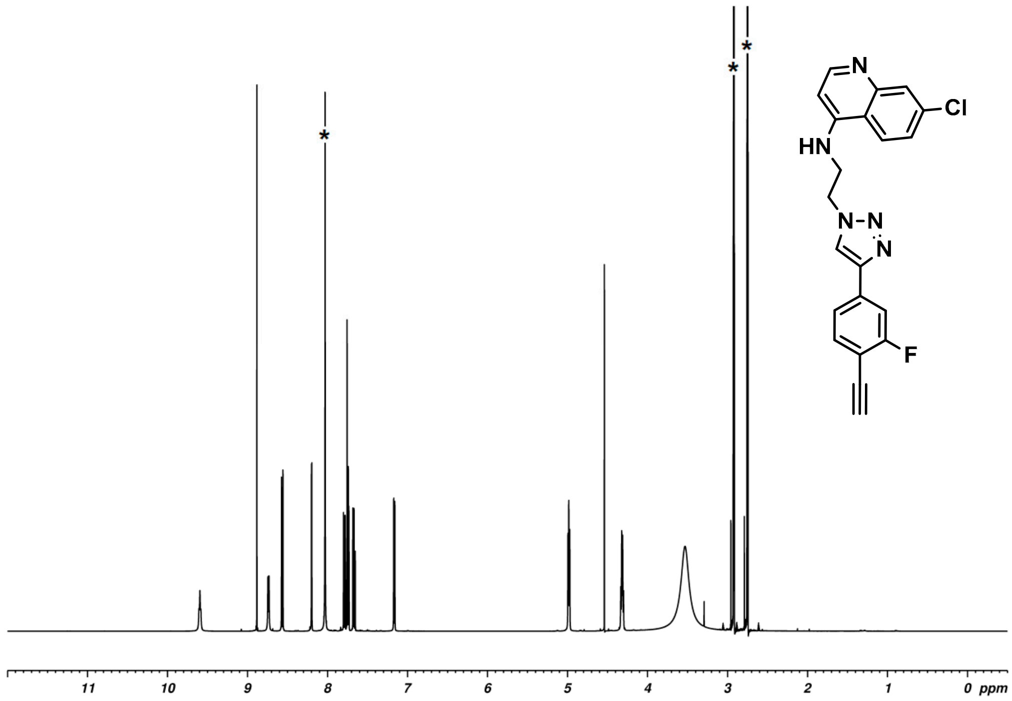

Figure S2. 500 MHz ^{1}H-NMR of compound **JR2** (in dDMF, * = solvent residual peak)

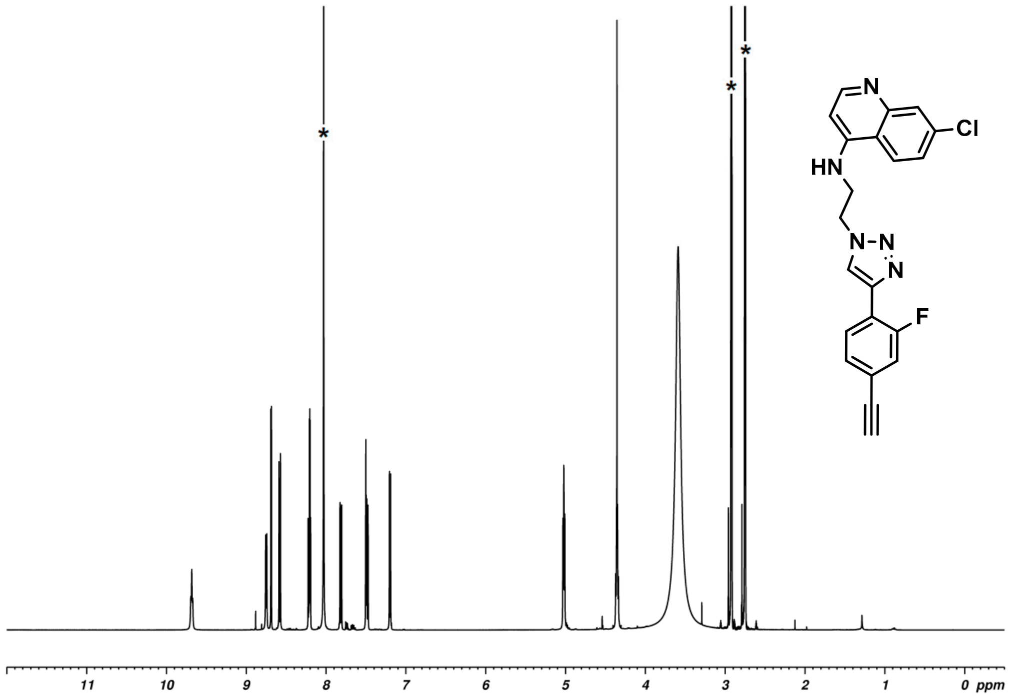

Figure S3. 500 MHz ^{1}H-NMR of compound **JR3** (in dDMF, * = solvent residual peak)

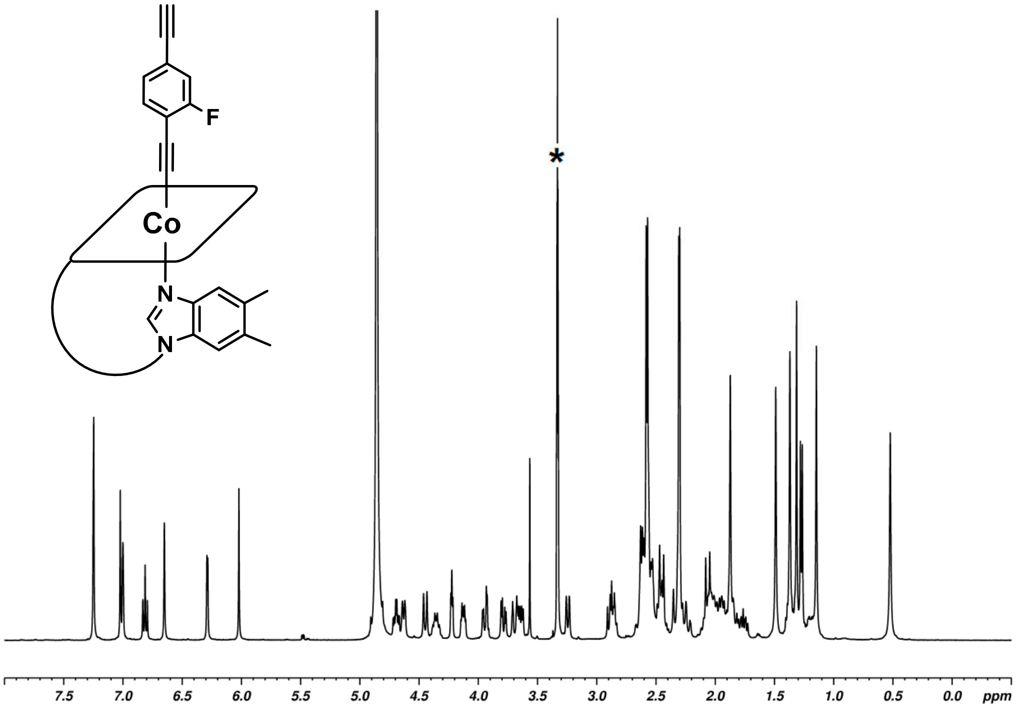

Figure S4. 500 MHz ^{1}H-NMR of compound B_{12}-**F1** (in MeOD, * = solvent residual peak)

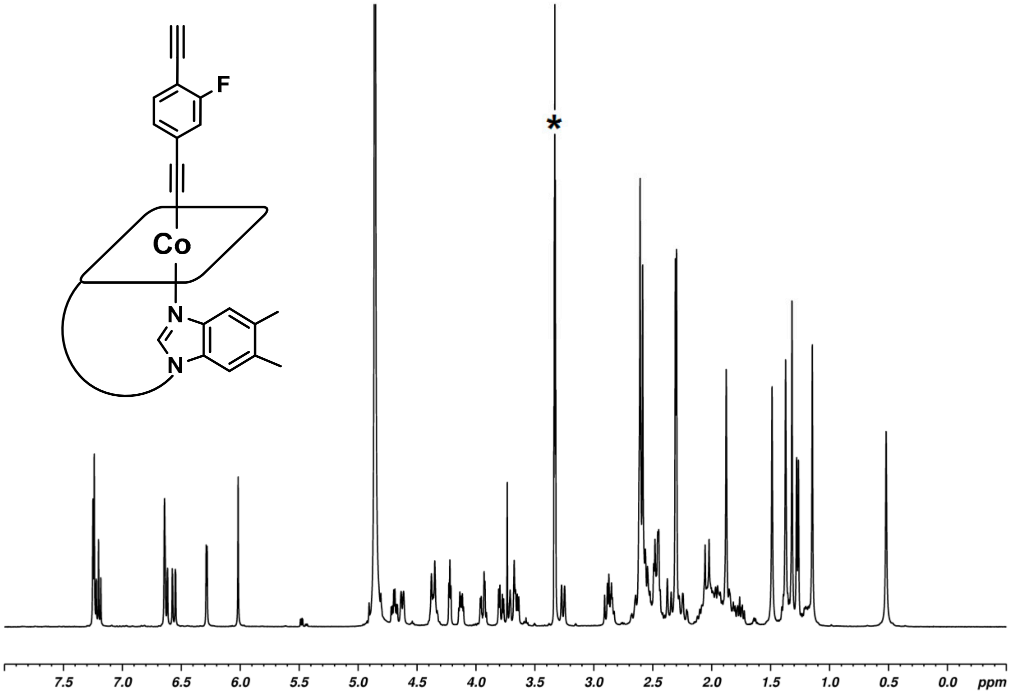

Figure S5. 500 MHz ^{1}H-NMR of compound B_{12}-**F2** (in MeOD, * = solvent residual peak)

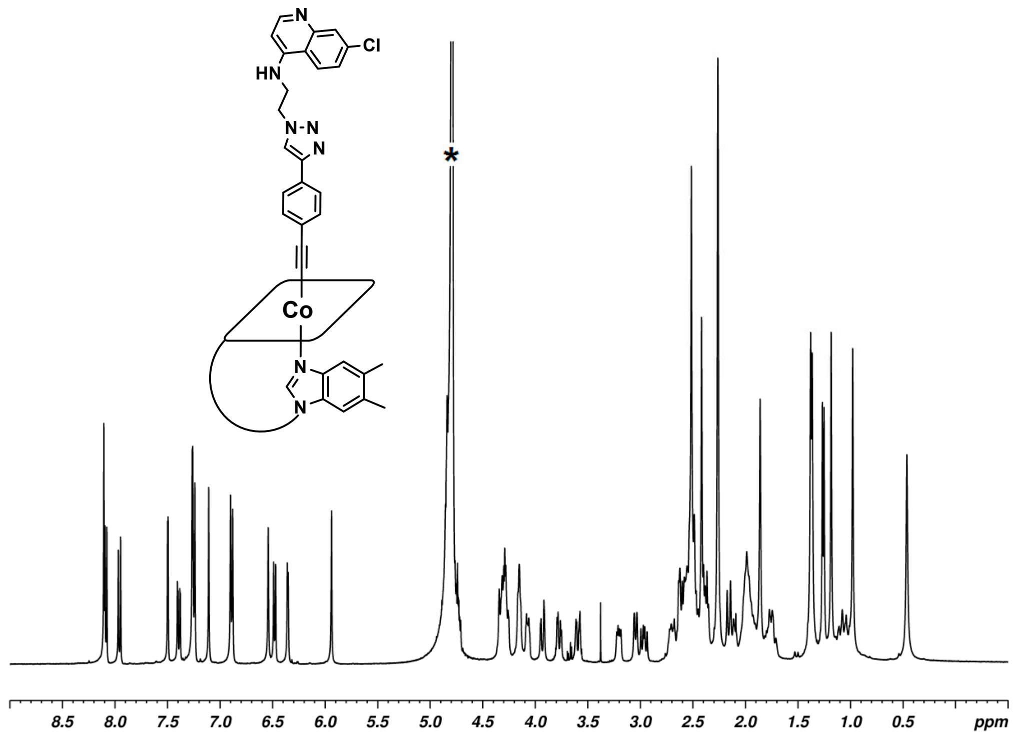

Figure S6. 500 MHz ^{1}H-NMR of compound B_{12}-**JR1** (in D_2O, * = solvent residual peak)

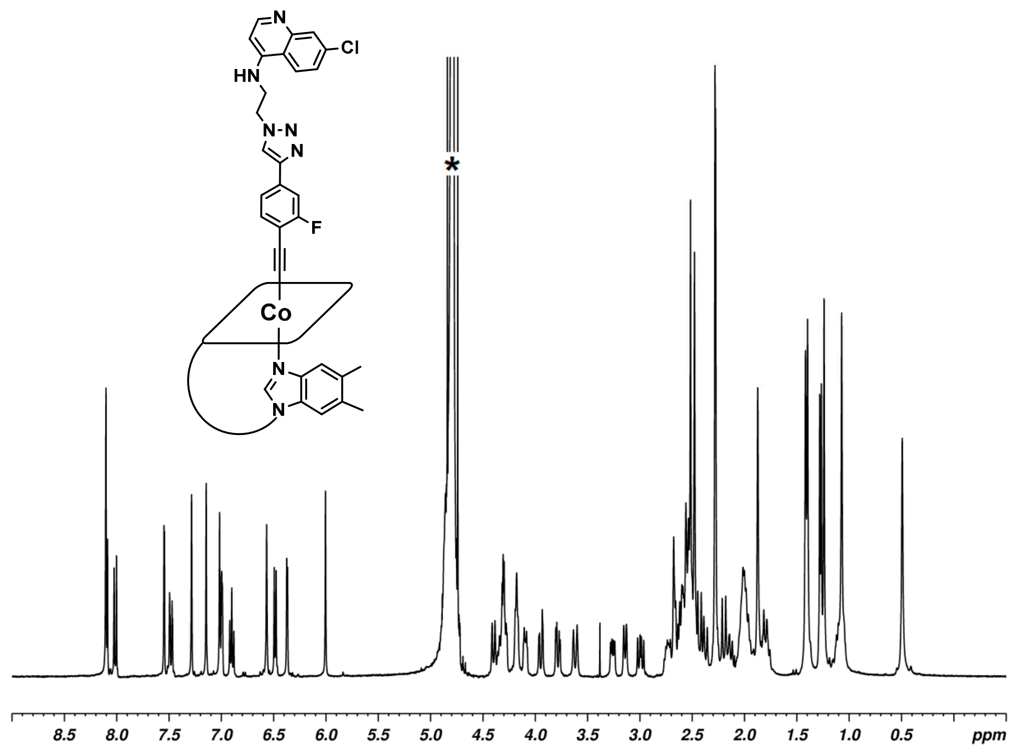

Figure S7. 500 MHz ^{1}H-NMR of compound B_{12}-**JR2** (in D_2O, * = solvent residual peak)

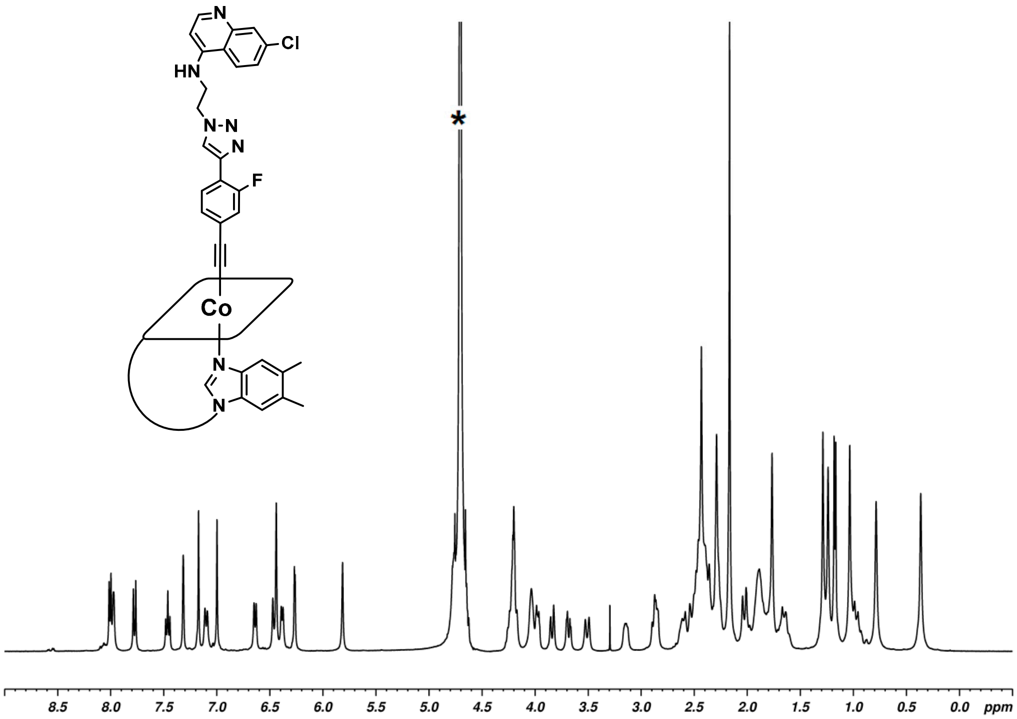

Figure S8 500 MHz ^{1}H-NMR of compound B_{12}-**JR3** (in D_2O, * = solvent residual peak)

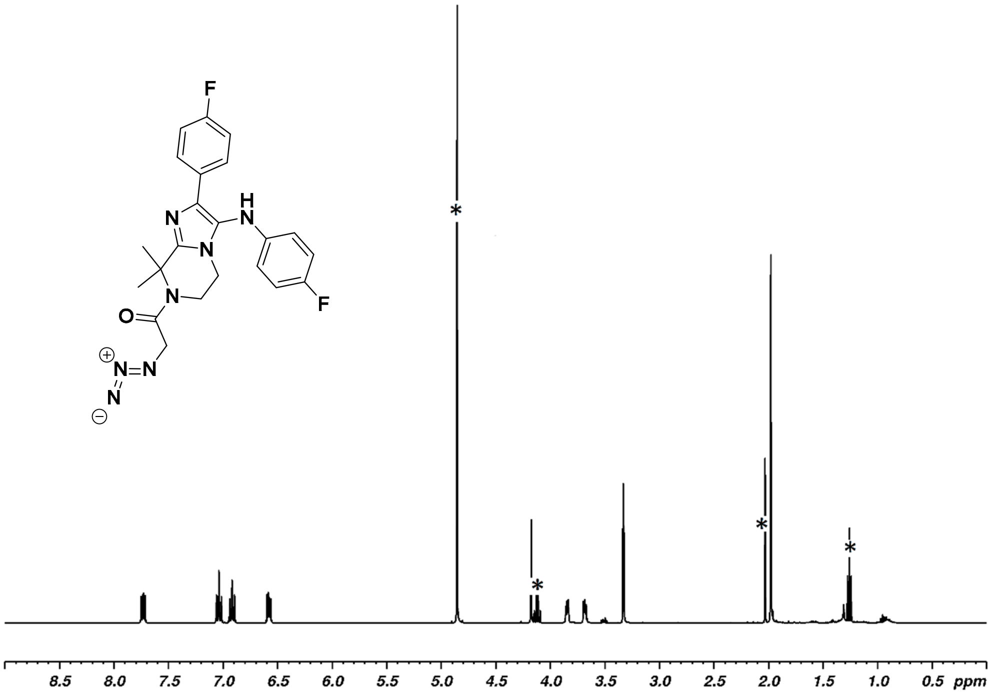

Figure S9 400 MHz ^{1}H-NMR of compound N_3-**SN1** (in CD_3OD, * = solvent residual peak)

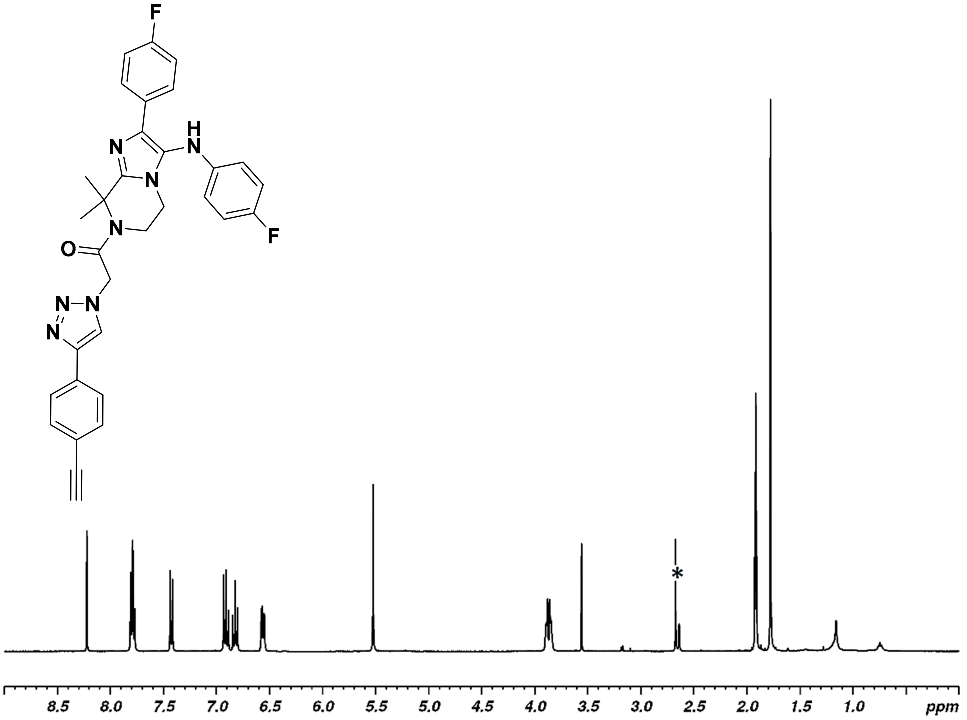

Figure S10 400 MHz ^{1}H-NMR of compound **SN1** (in $(CD_3)_2CO$, * = solvent residual peak)

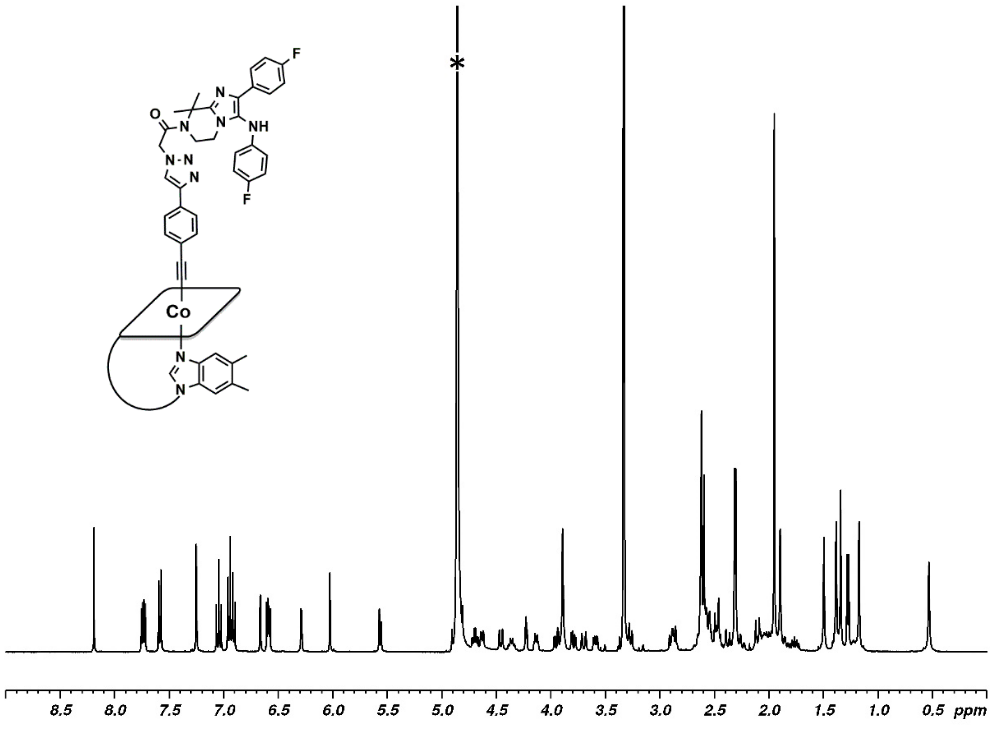

Figure S11 400 MHz ^{1}H-NMR of compound B_{12}-**SN1** (in D_2O, * = solvent residual peak)

Crystallographic details

Compound JR1

Table S1 Crystallographic details of compound JR1

Formula	$C_{23}H_{17}ClF_3N_5O_2$
$D_{calc.}$/ g cm^{-3}	1.535
μ/mm^{-1}	2.138
Formula Weight	487.86
Colour	colourless
Shape	prism
Size/mm^3	0.22×0.15×0.11
T/K	100.00(10)
Crystal System	triclinic
Space Group	$P\bar{1}$
a/Å	10.1159(4)
b/Å	10.3702(5)
c/Å	11.3845(6)
α/°	64.794(5)
β/°	89.752(4)
γ/°	78.740(4)
V/Å^3	1055.57(9)
Z	2
Z'	1
Wavelength/Å	1.54184
Radiation type	CuKα
Θ_{min}/°	4.309
Θ_{max}/°	76.236
Measured Refl.	9221
Independent Refl.	4328
Reflections with I > 2(I)	3887
R_{int}	0.0207
Parameters	463
Restraints	607
Largest Peak/e Å^{-3}	0.508
Deepest Hole/e Å^{-3}	-0.336
GooF	1.042
wR_2 (all data)	0.1134
wR_2	0.1096
R_1 (all data)	0.0465
R_1	0.0423

Experimental. Single colorless prism-shaped crystals of **JR1** were obtained by recrystallisation from **DCM/Hexane** at **Room temperature**. A suitable crystal of 0.22×0.15×0.11 mm^3 was selected and mounted on a suitable support on a SuperNova, Dual, Cu at zero, AtlasS2 diffractometer. The crystal was kept at a steady T = 100.00(10) K during data collection. The structure was solved with the **ShelXT** (Sheldrick, 2015[1]) program using the dual solution method and by using **Olex2** (Dolomanov et al., 2009[2]) as the graphical interface. The model was refined with version 2018/3 of ShelXL-2018/3 (Sheldrick, 2015[3]) using full matrix least squares on $|F|^2$ minimisation.

Crystal Data. $C_{23}H_{17}ClF_3N_5O_2$, M_r = 487.86, triclinic, P-1 (No. 2), a = 10.1159(4) Å, b = 10.3702(5) Å, c = 11.3845(6) Å, α = 64.794(5)°, β = 89.752(4)°, γ = 78.740(4)°, V = 1055.57(9) Å^3,

T = 100.00(10) K, Z = 2, Z' = 1, μ(CuKα) = 2.138, 9221 reflections measured, 4328 unique (R_{int} = 0.0207) which were used in all calculations. The final wR_2 was 0.1134 (all data) and R_1 was 0.0423 (I > 2(I)). A colorless prism-shaped crystal with dimensions of 0.22×0.15×0.11 mm^3 was mounted on a suitable support. Data were collected using a SuperNova, Dual, Cu at zero, AtlasS2 diffractometer operating at T = 100.00(10) K. Data were measured using ω scans using CuKα radiation. The total number of runs and images was based on the strategy calculation from the program **CrysAlisPro** (Rigaku, V1.171.38.46, 2015). The maximum resolution achieved was Θ = 76.236° (0.83 Å). The diffraction pattern was indexed. The total number of runs and images was based on the strategy calculation from the program **CrysAlisPro** (Rigaku, V1.171.38.46, 2015) and the unit cell was refined using **CrysAlisPro** (Rigaku, V1.171.38.46, 2015) on 5458 reflections, 59% of the observed reflections. Data reduction, scaling and absorption corrections were performed using **CrysAlisPro** (Rigaku, V1.171.38.46, 2015). The final completeness is 99.70 % out to 76.236° in Θ. A Gaussian absorption correction was performed using CrysAlisPro 1.171.38.46 (Rigaku Oxford Diffraction, 2015[4]) Numerical absorption correction based on Gaussian integration over a multifaceted crystal model/Empirical absorption correction using spherical harmonics as implemented in SCALE3 ABSPACK. The absorption coefficient μ of this material is 2.138 mm^{-1} at this wavelength (λ = 1.542Å) and the minimum and maximum transmissions are 0.583 and 1.000.

The structure was solved and the space group $P\bar{1}$ (# 2) determined by the **ShelXT** (Sheldrick, 2015[1]) structure solution program using dual and refined by full matrix least squares on $|F|^2$ using version 2018/3 of ShelXL-2018/3 (Sheldrick, 2015[3]). All non-hydrogen atoms were refined anisotropically. Hydrogen atom positions were calculated geometrically and refined using the riding model. Hydrogen atom positions were calculated geometrically and refined using the riding model. There is a single molecule in the asymmetric unit, which is represented by the reported sum formula. In other words: Z is 2 and Z' is 1.

1) Sheldrick, G.M., ShelXT-Integrated space-group and crystal-structure determination, *Acta Cryst.*, (2015), **A71**, 3-8.

2) O.V. Dolomanov and L.J. Bourhis and R.J. Gildea and J.A.K. Howard and H. Puschmann, Olex2: A complete structure solution, refinement and analysis program, *J. Appl. Cryst.*, (2009), **42**, 339-341.

3) Sheldrick, G.M., Crystal structure refinement with ShelXL, *Acta Cryst.*, (2015), **C27**, 3-8.

4) CrysAlisPro Software System, Rigaku Oxford Diffraction, (2015).

Compound B_{12}-F2

**Table S2** Crystallographic	details of compound B12-F2
Formula	$C_{74}H_{107}CoFN_{14}O_{20}P$
$D_{calc.}$/ g cm^{-3}	1.207
μ/mm^{-1}	2.286
Formula Weight	1621.63
Colour	red
Shape	prism
Size/mm^3	0.55×0.11×0.07
T/K	100.00(10)
Crystal System	orthorhombic
Flack Parameter	-0.0206(18)
Hooft Parameter	0.0066(12)
Space Group	$P2_12_12_1$
a/Å	15.8120(2)
b/Å	21.7525(4)
c/Å	25.9475(5)
α/°	90
β/°	90
γ/°	90
V/Å^3	8924.7(3)
Z	4
Z'	1
Wavelength/Å	1.54184
Radiation type	CuKα
Θ_{min}/°	2.651
Θ_{max}/°	76.013
Measured Refl.	34311
Independent Refl.	18085
Reflections with I > 2(I)	16718
R_{int}	0.0246
Parameters	1092
Restraints	260
Largest Peak/e Å^{-3}	0.950
Deepest Hole/e Å^{-3}	-0.444
GooF	1.035
wR_2 (all data)	0.1773
wR_2	0.1721
R_1 (all data)	0.0680
R_1	0.0632

Experimental. Single red prism-shaped crystals of **B_{12}-F2** were obtained by recrystallization from **Water/Acetonitrile** at **5°C**. A suitable crystal of 0.55×0.11×0.07 mm^3 was selected and mounted on a suitable support on a SuperNova, Dual, Cu at zero, AtlasS2 diffractometer. The crystal was kept at a steady T = 100.00(10) K during data collection. The structure was solved with the **ShelXT** (Sheldrick, 2015[1]) program using the dual solution method and by using **Olex2** (Dolomanov et al., 2009[2]) as the graphical interface. The model was refined with version 2018/3 of ShelXL-2018/3 (Sheldrick, 2015[3]) using full matrix least squares on $|F|^2$ minimisation.

Crystal Data. $C_{74}H_{107}CoFN_{14}O_{20}P$, M_r = 1621.63, orthorhombic, $P2_12_12_1$ (No. 19), a = 15.8120(2) Å,

b = 21.7525(4) Å, c = 25.9475(5) Å, $\alpha = \beta = \gamma = 90^{\circ}$, V = 8924.7(3) Å^3, T = 100.00(10) K, Z = 4, Z' = 1, μ (CuKα) = 2.286, 34311 reflections measured, 18085 unique (R_{int} = 0.0246) which were used in all calculations. The final wR_2 was 0.1773 (all data) and R_1 was 0.0632 (I > 2(I)). A red prism-shaped crystal with dimensions of 0.55×0.11×0.07 mm3 was mounted on a suitable support. Data were collected using a SuperNova, Dual, Cu at zero, AtlasS2 diffractometer operating at T = 100.00(10) K. Data were measured using ω scans using CuKα radiation. The total number of runs and images was based on the strategy calculation from the program CrysAlisPro (Rigaku, V1.171.38.46, 2015). The maximum resolution achieved was Θ = 76.013° (0.83 Å). The diffraction pattern was indexed. The total number of runs and images was based on the strategy calculation from the program CrysAlisPro (Rigaku, V1.171.38.46, 2015) and the unit cell was refined using CrysAlisPro (Rigaku, V1.171.38.46, 2015) on 14110 reflections, 41% of the observed reflections. Data reduction, scaling and absorption corrections were performed using CrysAlisPro (Rigaku, V1.171.38.46, 2015). The final completeness is 99.90 % out to 76.013° in Θ. A Gaussian absorption correction was performed using CrysAlisPro 1.171.38.46 (Rigaku Oxford Diffraction, 2015[4]) Numerical absorption correction based on Gaussian integration over a multifaceted crystal model/Empirical absorption correction using spherical harmonics as implemented in SCALE3 ABSPACK. The absorption coefficient μ of this material is 2.286 mm-1 at this wavelength (λ = 1.542Å) and the minimum and maximum transmissions are 0.488 and 1.000.

The structure was solved and the space group $P2_12_12_1$ (# 19) determined by the ShelXT (Sheldrick, 2015) structure solution program using dual and refined by full matrix least squares on $|\boldsymbol{F}|^2$ using version 2018/3 of ShelXL-2018/3 (Sheldrick, 2015). All non-hydrogen atoms were refined anisotropically. Hydrogen atom positions were calculated geometrically and refined using the riding model. A solvent mask was calculated and 224.0 electrons were found in a volume of 1120.0 Å^3 in two voids. This is consistent with the presence of two acetonitrile solvent molecules per formula unit which account for 256.0 electrons.

1) Sheldrick, G.M., ShelXT-Integrated space-group and crystal-structure determination, *Acta Cryst.*, (2015), **A71**, 3-8.

2) O.V. Dolomanov and L.J. Bourhis and R.J. Gildea and J.A.K. Howard and H. Puschmann, Olex2: A complete structure solution, refinement and analysis program, *J. Appl. Cryst.*, (2009), **42**, 339-341.

3) Sheldrick, G.M., Crystal structure refinement with ShelXL, *Acta Cryst.*, (2015), **C27**, 3-8.

4) CrysAlisPro Software System, Rigaku Oxford Diffraction, (2015).

DFT details and molecular coordinates

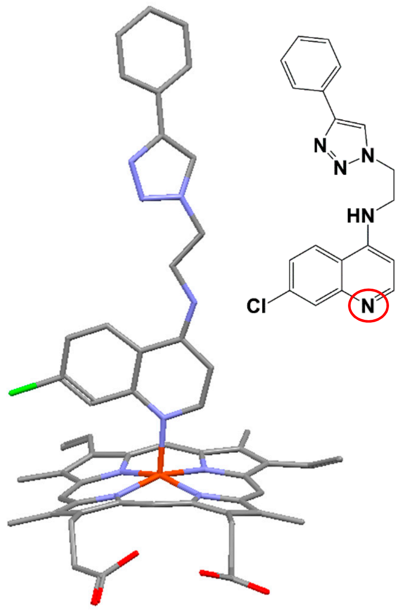

Figure S12 DFT optimized structure of N-quinoline bound JR1 model with ferriprotoporphyrin IX.

Atomic coordinates. B3LYP, LanL2DZ basis set

Fe	-2.290500	1.189100	-0.376000
N	-2.880400	-0.451400	-1.366800
C	-3.093600	-0.608500	-2.742100
C	-3.029700	-1.730300	-0.824200
C	-3.405200	-1.993000	-3.060200
C	-3.332400	-2.697900	-1.871900
N	-2.231900	2.166700	-2.131300
C	-2.460300	1.671500	-3.421100
C	-1.778100	3.475200	-2.315600
C	-2.138900	2.684800	-4.424200
C	-1.712200	3.807200	-3.727100
N	-2.286200	2.949600	0.562400
C	-1.828000	4.164100	0.044300
C	-2.632400	3.233300	1.895900
C	-1.864700	5.211300	1.062700
C	-2.393300	4.628900	2.211600
N	-2.876600	0.323600	1.308100
C	-3.121700	0.915900	2.556300
C	-2.993500	-1.054100	1.534400
C	-3.375300	-0.096100	3.568600
C	-3.264500	-1.324200	2.941700
C	-3.009500	-2.023700	0.537800
C	-3.062100	2.279800	2.814100
C	-1.531800	4.388500	-1.294200
C	-2.931200	0.391700	-3.696000
C	-3.725500	-2.513900	-4.435800
H	-4.681900	-2.117500	-4.803500
H	-2.956700	-2.235500	-5.168700
H	-3.800600	-3.605900	-4.440100
C	-3.675800	0.192600	5.014400
H	-2.871300	0.773100	5.485300
H	-4.606000	0.766000	5.127300
H	-3.792400	-0.732600	5.587000
C	-2.633500	5.269900	3.550000
H	-3.600000	5.792800	3.579900
H	-2.632600	4.538600	4.364200

H	-1.855400	6.010200	3.769500
C	-1.265700	5.132700	-4.281200
H	-1.945100	5.943400	-3.983800
H	-0.261600	5.401100	-3.926300
H	-1.228300	5.114600	-5.374200
C	-1.398200	6.580800	0.833300
H	-0.674100	6.705900	0.027800
C	-1.763900	7.695400	1.513900
H	-2.505300	7.687900	2.306400
H	-1.334200	8.661900	1.264100
C	-2.252200	2.575000	-5.887000
H	-2.572600	3.486300	-6.393800
C	-1.974100	1.496800	-6.658300
H	-1.590700	0.564000	-6.252300
H	-2.094100	1.541300	-7.737500
C	-3.555500	-4.174800	-1.664200
H	-3.126500	-4.735400	-2.504500
H	-3.043200	-4.516700	-0.759000
C	-5.073900	-4.535500	-1.547400
H	-5.517900	-3.948100	-0.736700
H	-5.594700	-4.306400	-2.479900
C	-3.441400	-2.695100	3.546500
H	-2.828800	-3.421100	3.005100
H	-3.068500	-2.683700	4.577000
C	-4.928000	-3.189200	3.559000
H	-5.558500	-2.483500	2.999700
H	-5.327200	-3.228900	4.576400
C	-5.248700	-5.999800	-1.216000
C	-5.166200	-4.555400	2.933500
O	-5.691900	-6.749500	-2.272700
H	-5.791600	-7.701600	-2.035500
O	-4.419100	-4.746100	1.786000
H	-4.648900	-5.575400	1.265100
O	-5.977600	-5.393700	3.350600
O	-4.995700	-6.523500	-0.108900
C	0.616400	0.759900	0.596500
C	0.093500	0.222000	-1.633000
C	0.220100	1.254700	1.866900
C	1.993500	0.339600	0.409500
C	1.393400	-0.187800	-1.896000
H	-0.636400	0.169300	-2.425100
C	1.123700	1.318000	2.910400
H	-0.792900	1.581700	2.007700
C	2.876600	0.426900	1.532100
C	2.398500	-0.147000	-0.899000
H	1.627700	-0.542700	-2.895300
C	2.464700	0.902600	2.768700
H	3.904400	0.113200	1.449000
H	3.154300	0.954600	3.603700
N	-0.329500	0.692600	-0.434200
Cl	0.576100	1.953200	4.516700
N	3.651500	-0.566300	-1.273600
H	3.710900	-0.874000	-2.239300
C	4.913800	-0.638400	-0.516800
H	5.211100	0.352100	-0.152500
H	4.828700	-1.311400	0.340300
C	6.020500	-1.175400	-1.448100
H	6.152900	-0.508900	-2.309700
H	5.748200	-2.174100	-1.819000
N	7.288800	-1.246600	-0.720000
C	8.578000	-1.197000	-1.191600
C	9.394200	-1.372800	-0.069300
H	8.813300	-1.047600	-2.232100
N	7.285000	-1.454400	0.665200
N	8.558900	-1.523800	1.042400
C	10.860100	-1.406200	0.047600
C	11.689500	-1.226200	-1.083900
C	11.457700	-1.622500	1.311300
C	13.087700	-1.262300	-0.955000
H	11.251400	-1.057200	-2.065700
C	12.855900	-1.658100	1.437200
H	10.818400	-1.760700	2.177900
C	13.677000	-1.478400	0.306300
H	13.715300	-1.122900	-1.831600
H	13.304900	-1.825700	2.412800
H	14.759000	-1.506400	0.405600

H	-3.150800	0.147700	-4.727600
H	-1.203000	5.379800	-1.583000
H	-3.300500	2.610400	3.817300
H	-3.153700	-3.053400	0.845400

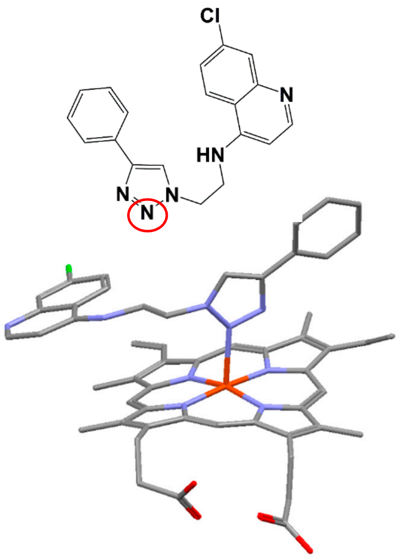

Figure S13 DFT optimized structure of N-triazole bound JR1 model with ferriprotoporphyrin IX.

Atomic coordinates. M06, LanL2DZ basis set

Fe	0.536000	-0.252200	0.907300
N	2.520600	-0.308900	0.787300
C	3.358600	-1.335500	1.208200
C	3.358600	0.643400	0.246500
C	4.759000	-0.975300	1.003600
C	4.756600	0.252300	0.393600
N	0.533800	-2.060800	1.650900
C	1.600100	-2.933800	1.760700
C	-0.586500	-2.814200	1.993900
C	1.136800	-4.292100	2.111000
C	-0.218200	-4.207400	2.270700
N	-1.370300	-0.021800	1.465900
C	-2.212100	-0.985700	1.976600
C	-2.134600	1.151300	1.444900
C	-3.519200	-0.415700	2.325500
C	-3.453000	0.929000	2.007200
N	0.572200	1.607900	0.286400
C	-0.459900	2.536000	0.317700
C	1.640100	2.282200	-0.322300
C	-0.062000	3.789100	-0.337300
C	1.221500	3.620200	-0.770400
C	2.937200	1.826100	-0.371800
C	-1.708100	2.348900	0.888500
C	-1.866300	-2.327900	2.148900
C	2.936200	-2.575200	1.653100
C	5.921600	-1.825200	1.393600
H	5.936700	-2.009800	2.474800
H	5.887900	-2.803400	0.897300

H	6.870900	-1.352400	1.124400
C	-0.932100	4.997300	-0.426000
H	-1.935600	4.750200	-0.799000
H	-1.064100	5.457700	0.561800
H	-0.499600	5.757500	-1.083800
C	-4.490700	1.978700	2.210700
H	-4.770000	2.047800	3.270400
H	-4.155800	2.970300	1.891900
H	-5.407600	1.754600	1.649200
C	-1.191400	-5.276700	2.633600
H	-1.696400	-5.052600	3.582000
H	-1.968600	-5.385100	1.866600
H	-0.699600	-6.247300	2.738700
C	-4.595000	-1.191100	2.915400
H	-4.352300	-2.217000	3.190400
C	-5.860600	-0.778000	3.162200
H	-6.224700	0.217500	2.927000
H	-6.583900	-1.452200	3.610100
C	1.958600	-5.490300	2.254000
H	1.607500	-6.215500	2.988400
C	3.063800	-5.794100	1.539200
H	3.443300	-5.155000	0.744100
H	3.598900	-6.722700	1.711400
C	5.930300	1.107600	0.040300
H	6.776000	0.484300	-0.275300
H	5.700100	1.770500	-0.803600
C	6.347100	1.963000	1.261800
H	5.479900	2.538000	1.618900
H	6.711900	1.334900	2.078800
C	2.070300	4.621600	-1.490700
H	2.767500	4.140600	-2.187200
H	1.418900	5.260900	-2.098400
C	2.876400	5.539000	-0.532700
H	2.266300	5.834600	0.325900
H	3.183700	6.435100	-1.081600
C	7.391900	2.966600	0.884500
C	4.125300	4.861000	-0.032200
O	8.491800	2.940700	1.672200
H	9.148000	3.628600	1.419700
O	4.993200	4.615800	-1.048700
H	5.897100	4.287700	-0.745100
O	4.348000	4.532200	1.143800
O	7.297700	3.766500	-0.066200
C	-6.147800	2.216400	-0.932600
C	-5.354600	4.214500	-1.767500
C	-7.229600	1.533600	-0.305100
C	-4.950000	1.495600	-1.287700
C	-4.144800	3.616300	-2.166200
H	-5.521500	5.271300	-1.958200
C	-7.124500	0.189500	-0.044400
H	-8.111300	2.115300	-0.057800
C	-4.914300	0.097600	-0.999600
C	-3.900600	2.255600	-1.925400
H	-3.399500	4.217100	-2.683600
C	-5.972000	-0.551200	-0.392300
H	-4.052300	-0.504100	-1.250700
H	-5.929800	-1.614500	-0.180500
N	-6.340800	3.558600	-1.155800
Cl	-8.472400	-0.674800	0.777100
N	-2.671400	1.760900	-2.333500
H	-2.084700	2.441900	-2.805500
C	-2.090200	0.445200	-2.136900
H	-2.101900	0.154400	-1.074600
H	-2.619700	-0.329300	-2.713500
C	-0.630300	0.478200	-2.598900
H	-0.065700	1.228900	-2.034900
H	-0.560700	0.710200	-3.667900
N	-0.016200	-0.826600	-2.382400

C	0.247700	-1.809200	-3.290000
C	0.739800	-2.884700	-2.547800
H	0.064300	-1.682900	-4.344700
N	0.286300	-1.261400	-1.111900
N	0.744700	-2.508600	-1.218500
C	1.198200	-4.203900	-2.982100
C	1.412400	-4.475900	-4.345600
C	1.432500	-5.213100	-2.029600
C	1.854000	-5.738900	-4.752400
H	1.248300	-3.700300	-5.090900
C	1.875600	-6.474700	-2.438500
H	1.255800	-4.999900	-0.977800
C	2.087800	-6.741800	-3.799800
H	2.019600	-5.941800	-5.805600
H	2.048600	-7.251700	-1.699800
H	2.430400	-7.721900	-4.116100
H	3.685700	-3.314600	1.914300
H	-2.633900	-3.021100	2.475700
H	-2.400200	3.185600	0.877100
H	3.690100	2.447800	-0.849500

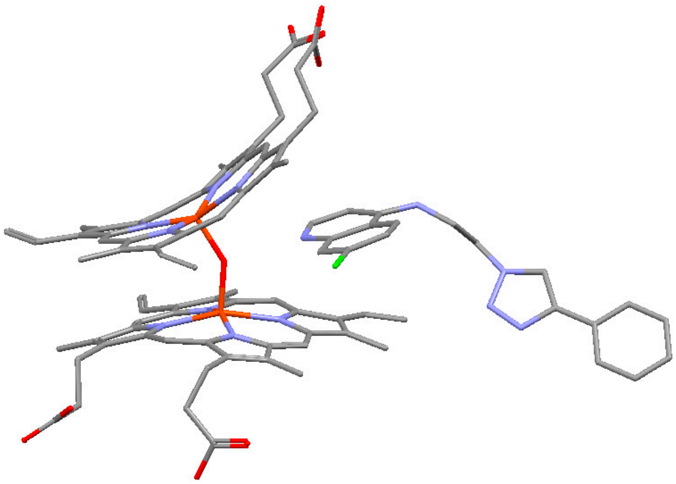

Figure S14 DFT optimized structure of N-quinoline protonated JR1 model interacting with ferriprotoporphyrin IX µ-oxo dimer.

Atomic coordinates. B3LYP, LanL2DZ basis set

Fe	-2.027100	1.588000	0.839000
O	-1.236300	0.346900	-0.163400
N	-1.075600	3.198300	0.089300
N	-0.681100	1.605800	2.372700
N	-3.207500	0.471600	1.996900
N	-3.647100	2.150300	-0.222100
C	-1.369000	3.821800	-1.127300
C	-0.373500	4.841000	-1.438600
C	0.493500	4.885600	-0.361700
C	0.056700	3.856500	0.575900
C	0.696400	3.572100	1.779300
H	1.522400	4.205100	2.084200

C	0.349800	2.519900	2.616400
C	1.042200	2.203300	3.863200
C	0.431600	1.078500	4.380500
C	-0.640700	0.724300	3.451300
C	-1.579100	-0.273900	3.695500
H	-1.430300	-0.897900	4.567500
C	-2.804000	-0.364700	3.040500
C	-3.949900	-1.113700	3.570200
C	-5.069300	-0.634000	2.905300
C	-4.594200	0.314100	1.914100
C	-5.424500	0.986200	1.026200
H	-6.490600	0.820100	1.121600
C	-4.983000	1.837800	0.022300
C	-5.862600	2.531400	-0.920900
C	-5.033300	3.226900	-1.790300
C	-3.670300	2.998200	-1.328800
C	-2.562600	3.678900	-1.825600
H	-2.691200	4.266000	-2.727500
C	-0.385900	5.712500	-2.665900
H	0.535400	6.299100	-2.750400
H	-1.223500	6.424100	-2.647600
H	-0.487700	5.117700	-3.582700
C	0.714900	0.370300	5.677700
H	1.636500	0.740200	6.139200
H	-0.097500	0.526100	6.401400
H	0.823100	-0.712800	5.541700
C	-3.950800	-2.112100	4.644200
H	-4.875700	-2.173600	5.219100
C	-2.953500	-2.970200	4.973700
H	-2.015900	-3.021000	4.426200
H	-3.073300	-3.668700	5.797900
C	-6.515100	-0.979700	3.143700
H	-6.619300	-1.807500	3.851600
H	-7.026500	-1.274800	2.219100

H	-7.064300	-0.121300	3.555300
C	-7.324900	2.447400	-0.884500
H	-7.738400	1.572600	-0.381300
C	-8.212400	3.341400	-1.387100
H	-7.910300	4.269500	-1.862100
H	-9.282100	3.168000	-1.302200
C	-5.416500	4.087400	-2.961600
H	-6.338400	3.721400	-3.428700
H	-4.638100	4.101500	-3.731000
H	-5.601200	5.128300	-2.657000
Fe	-1.509200	-0.879500	-1.427400
O	-1.793300	-7.837100	1.140300
O	-3.500400	-7.980700	-0.390500
N	-1.386900	-2.554900	-0.330600
N	-3.521100	-1.122000	-1.451900
N	-1.675800	0.285100	-3.061200
N	0.487200	-1.139600	-1.918200
C	-0.250100	-3.322800	-0.039400
C	-0.572600	-4.414700	0.876400
C	-1.918800	-4.305400	1.159500
C	-2.414700	-3.159300	0.398600
C	-3.760300	-2.830200	0.305800
H	-4.457400	-3.385100	0.922500
C	-4.281400	-1.945500	-0.629800
C	-5.697100	-1.890300	-0.994200
C	-5.793800	-1.031200	-2.066200
C	-4.440100	-0.552100	-2.334000
C	-4.125600	0.314600	-3.367900
H	-4.948200	0.696900	-3.959800
C	-2.837600	0.717900	-3.699300
C	-2.526500	1.645200	-4.785000
C	-1.143400	1.788100	-4.795900
C	-0.630100	0.925200	-3.743200
C	0.725000	0.663700	-3.570300

H	1.414300	1.219500	-4.193400
C	1.244900	-0.359000	-2.784500
C	2.632800	-0.828900	-2.843500
C	2.700000	-1.946000	-2.012800
C	1.371600	-2.114400	-1.447300
C	1.019700	-3.125300	-0.557500
H	1.787500	-3.827400	-0.255900
C	0.414300	-5.447500	1.351100
H	-0.081900	-6.237400	1.919000
H	1.193800	-5.000500	1.982200
H	0.913300	-5.938000	0.504600
C	-2.778800	-5.202500	2.019200
H	-3.448900	-4.593800	2.638100
H	-2.143800	-5.766600	2.711200
C	-3.647400	-6.213400	1.224000
H	-4.424400	-6.628700	1.882800
H	-4.175400	-5.741300	0.390900
C	-2.859800	-7.391800	0.689100
C	-6.781100	-2.739200	-0.383300
H	-6.570500	-2.948200	0.670500
H	-7.742700	-2.210700	-0.418900
C	-6.937100	-4.102500	-1.130600
H	-7.269600	-3.937600	-2.159100
H	-5.966900	-4.612200	-1.158700
C	-7.940400	-5.008000	-0.453500
O	-9.033300	-5.377100	-0.901200
O	-7.497900	-5.400700	0.812800
C	-7.011500	-0.675000	-2.874600
H	-7.916600	-1.123100	-2.450800
H	-6.925500	-1.032500	-3.910100
H	-7.169800	0.410200	-2.914400
C	-3.531100	2.210600	-5.687000
H	-4.495900	1.703700	-5.693000
C	-3.402200	3.275100	-6.520000

H	-2.500200	3.874300	-6.586500
H	-4.227400	3.575900	-7.160300
C	-0.311900	2.587800	-5.761400
H	-0.701600	2.487300	-6.782300
H	0.733800	2.266500	-5.777400
H	-0.319800	3.661100	-5.520000
C	3.658100	-0.226400	-3.694000
H	3.313200	0.582000	-4.339300
C	4.973400	-0.555400	-3.799300
H	5.445300	-1.348500	-3.229900
H	5.606600	-0.033100	-4.513800
C	3.872500	-2.859800	-1.783600
H	4.732300	-2.341700	-1.338500
H	4.227500	-3.283100	-2.733600
H	3.622700	-3.700200	-1.129900
H	-3.010200	-8.775300	-0.705100
H	-8.157400	-5.993700	1.241300
C	2.188200	3.008000	4.426500
H	2.906000	3.225700	3.628000
H	2.723700	2.412200	5.174000
C	1.650600	5.828100	-0.141500
H	2.084600	6.120700	-1.106300
H	2.442600	5.339000	0.437100
C	1.215300	7.126200	0.619200
H	0.740900	6.839900	1.564000
H	0.499000	7.694300	0.021300
C	1.752100	4.360900	5.084800
H	0.708700	4.578200	4.817000
H	1.796100	4.306800	6.176000
C	2.409400	7.995200	0.937800
C	2.561900	5.575000	4.655800
O	2.470200	9.136600	0.180600
H	3.254700	9.689000	0.408700
O	2.779500	5.594100	3.288400

H	3.156200	6.448700	2.921200
O	2.945500	6.484700	5.404600
O	3.297700	7.731500	1.778600
Cl	1.927500	-2.999200	4.045500
C	2.616200	-1.729200	2.951700
C	1.788700	-1.101400	2.038100
C	3.984700	-1.396600	3.092300
C	2.344400	-0.105300	1.194100
H	0.734500	-1.339800	1.969800
C	4.531600	-0.441500	2.245100
H	4.588000	-1.876300	3.855200
C	3.744300	0.204700	1.245400
H	5.568300	-0.162900	2.384400
C	4.266000	1.204600	0.329300
C	1.987700	1.602000	-0.430600
C	3.338400	1.939200	-0.441400
N	5.612200	1.471200	0.190100
H	1.267400	2.103900	-1.060500
H	3.676600	2.713700	-1.121200
H	5.829700	2.328500	-0.310000
C	6.710800	0.484600	0.238300
H	7.383500	0.674300	1.084800
H	6.290400	-0.516600	0.343300
C	7.509300	0.508200	-1.093300
H	8.185300	1.368600	-1.145000
H	6.805200	0.558600	-1.931700
N	8.297700	-0.716200	-1.231500
C	9.653500	-0.931200	-1.214900
C	9.815900	-2.311500	-1.380800
H	10.369100	-0.134200	-1.100900
N	8.541900	-2.877900	-1.489700
C	11.039500	-3.125800	-1.446400
C	12.319500	-2.534900	-1.332900
C	10.941900	-4.525400	-1.626400
C	13.478000	-3.326600	-1.397800
H	12.419200	-1.459900	-1.195400
C	12.101800	-5.314500	-1.690900
H	9.958300	-4.976700	-1.714800
C	13.374100	-4.720100	-1.577000
H	14.456400	-2.861100	-1.309800
H	12.015200	-6.389100	-1.830100
H	0.492000	0.379400	0.257300
N	1.517500	0.595000	0.331600
H	14.270900	-5.332300	-1.627500
N	7.615100	-1.924700	-1.400800

Additional supporting images

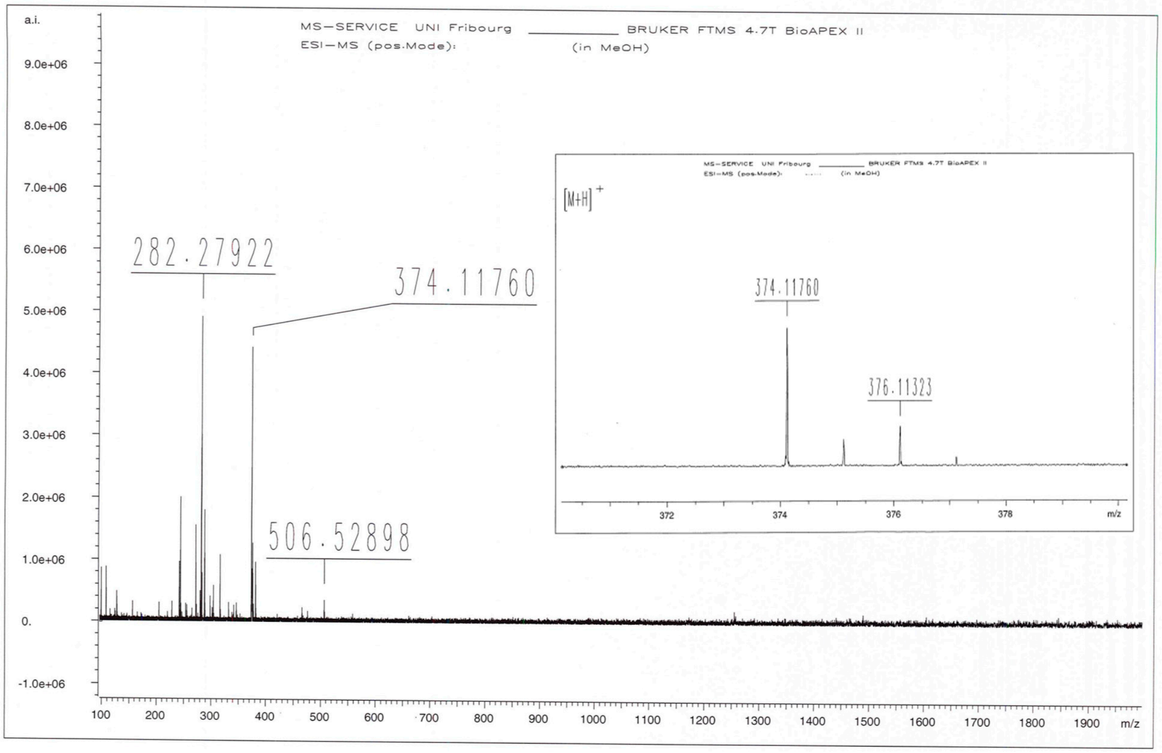

Figure S15. HR-ESI-MS spectrum (in MeOH) of compound **JR1**

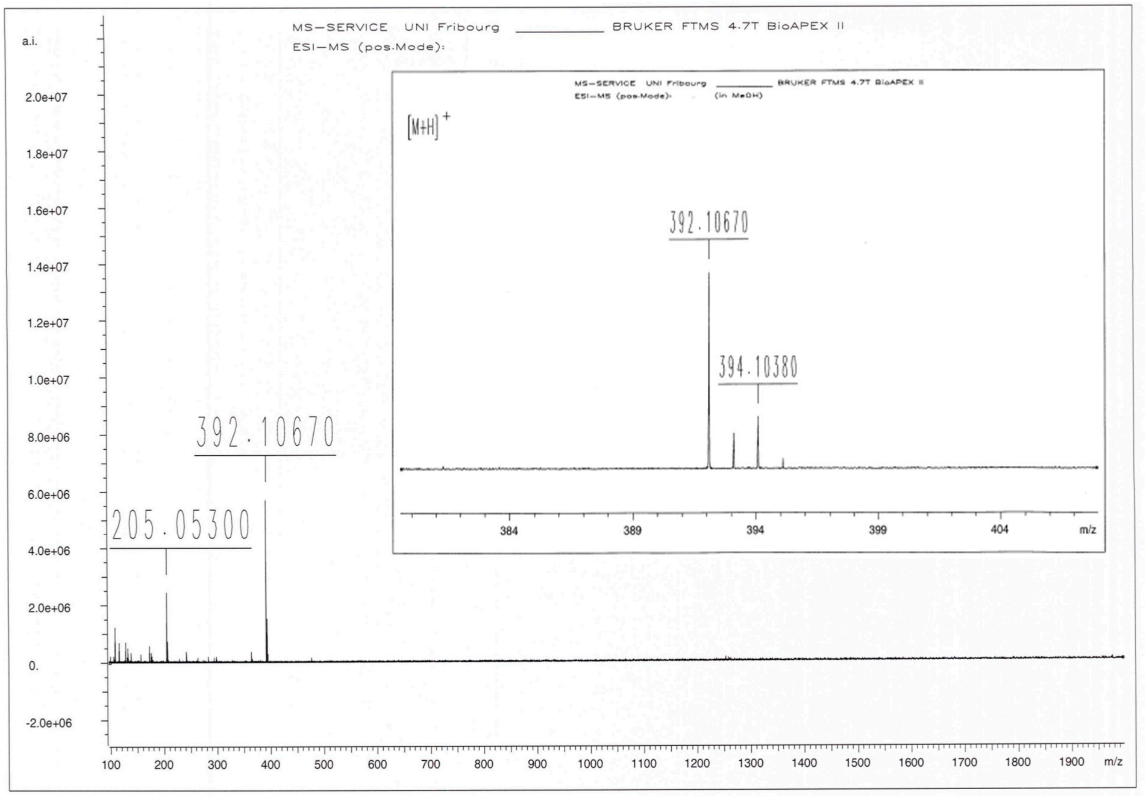

Figure S16. HR-ESI-MS spectrum (in MeOH) of compound **JR2/3**

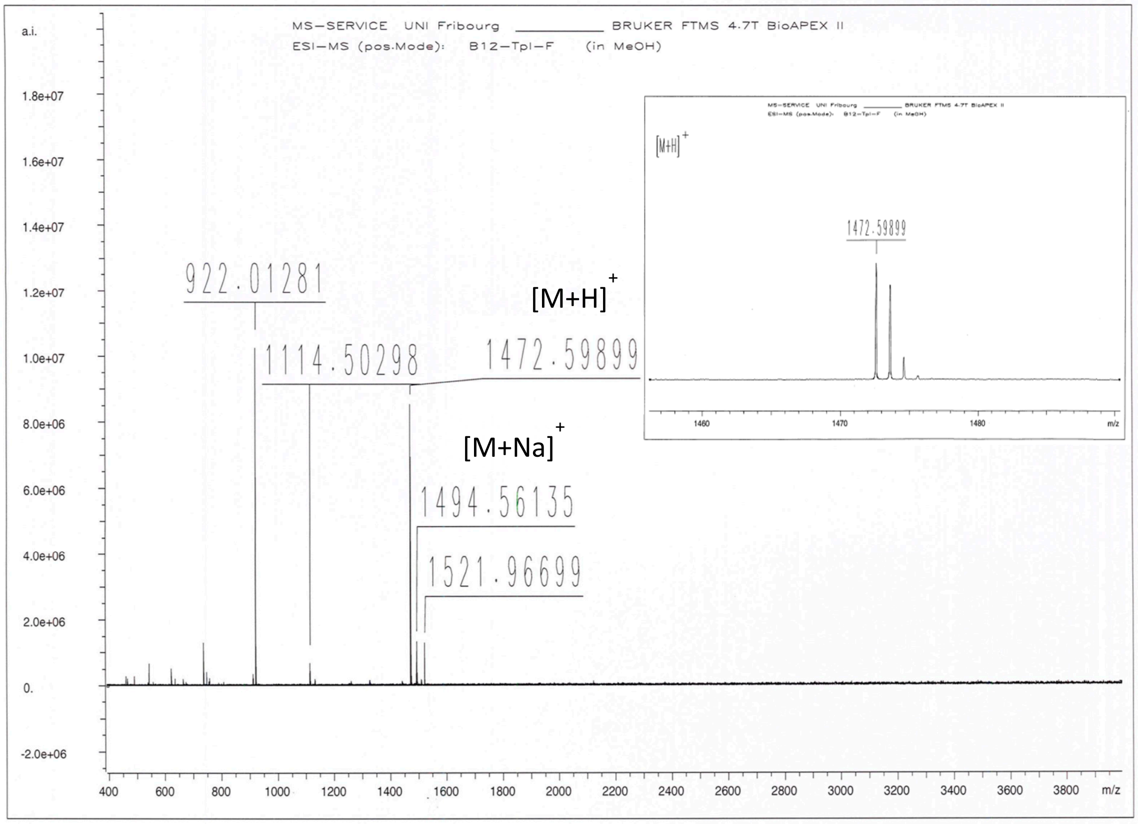

Figure S17. HR-ESI-MS spectrum (in MeOH) of compound B_{12}-**F1/2**

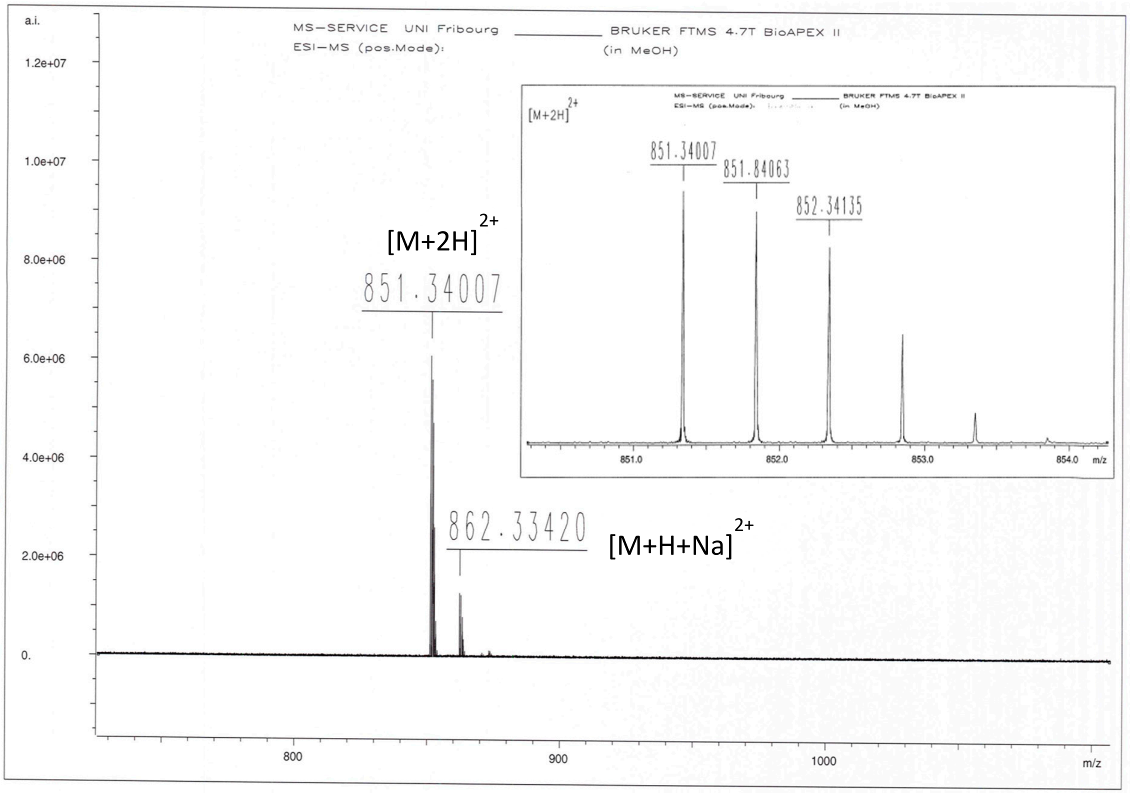

Figure S18. HR-ESI-MS spectrum (in MeOH) of compound B_{12}-**JR1**

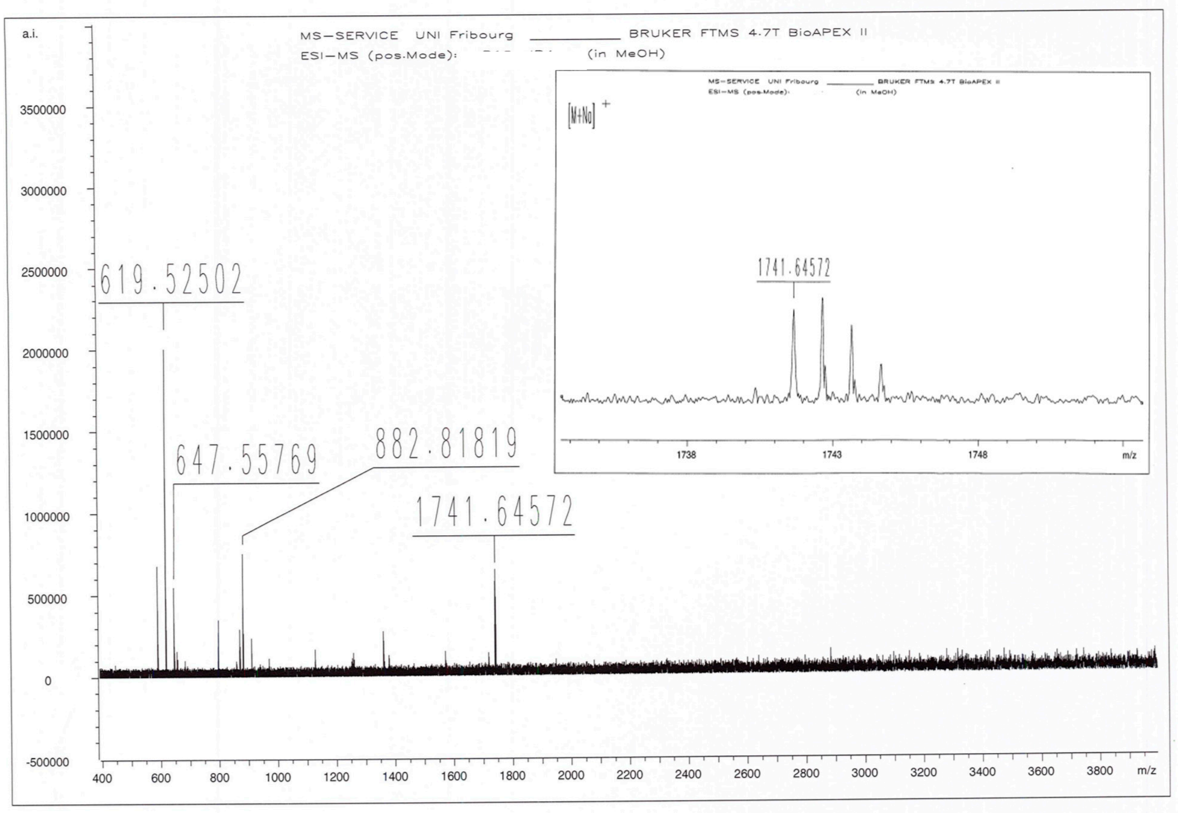

Figure S19. HR-ESI-MS spectrum (in MeOH) of compound B_{12}-**JR2/3**

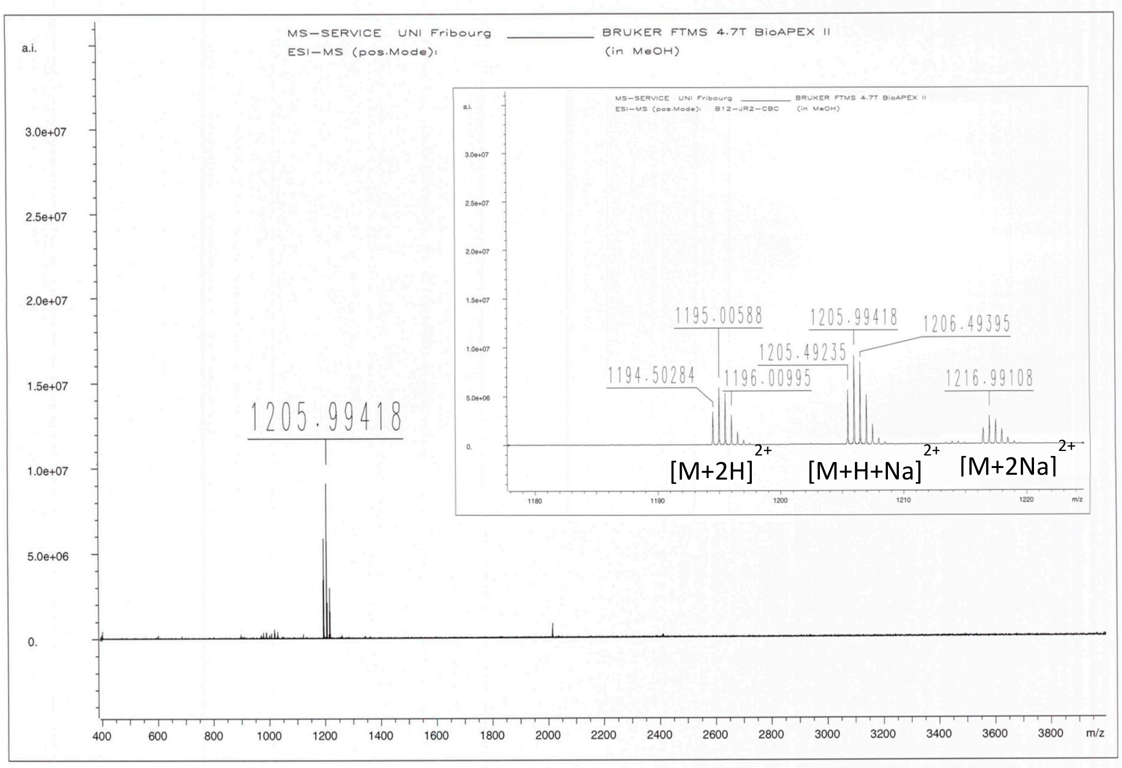

Figure S20. HR-ESI-MS spectrum (in MeOH) of compound B_{12}-**JR1-CBC**

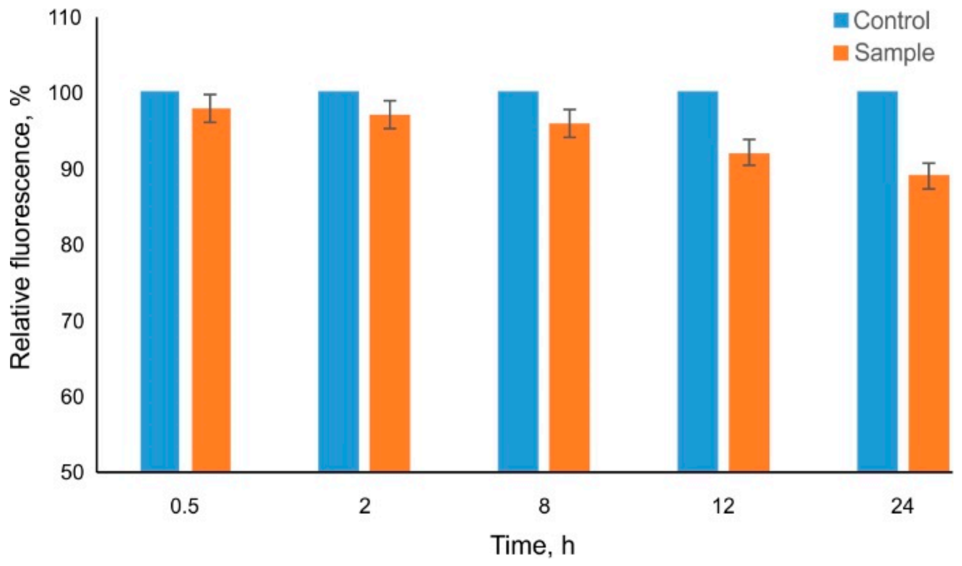

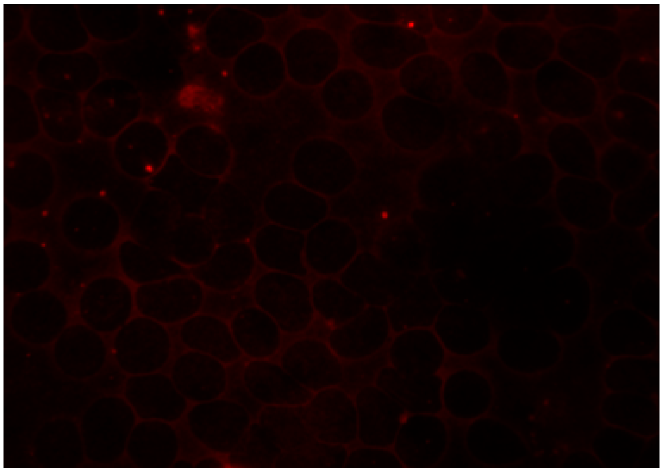

Figure S21. Top: distribution of compound B12-**JR1-CBC** in suspension of washed red blood cells (RBC) over time. RBC suspensions with (Sample) and without (Control) the molecule were incubated with 20 μM (final concentration) of B12-**JR1-CBC** at 37°C for 24 h in dark with shaking and at various time points aliquots were centrifuged and the amount of fluorescence in the supernatant determined (λEx = 488 nm, λEm = 540 nm). Bottom: fluorescence spectra of full smear blood control.

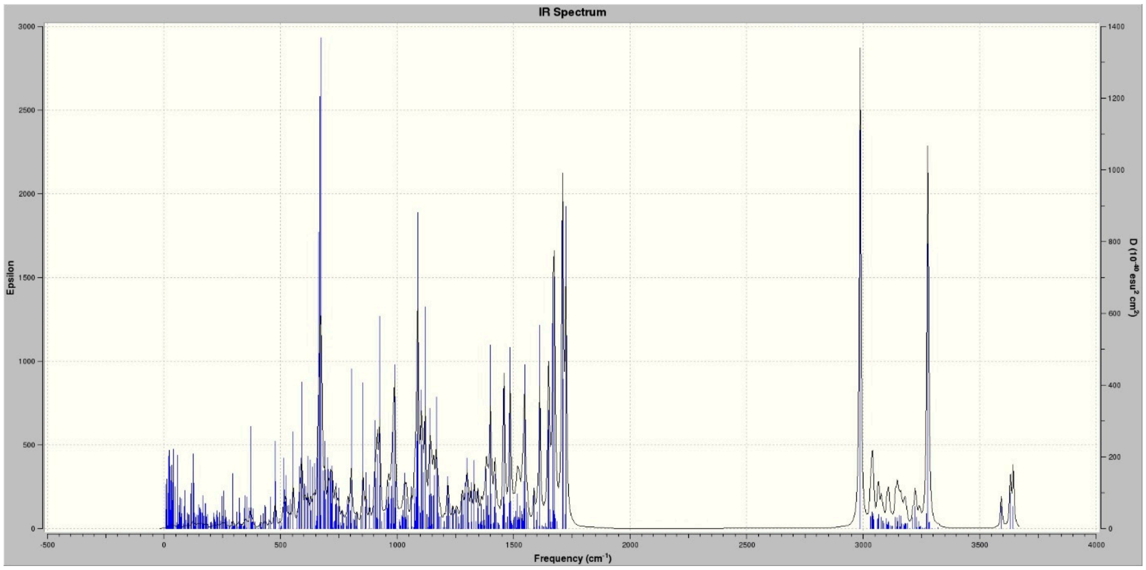

Figure S22. Calculated IR spectrum of DFT optimized structure (gas-phase) of the interaction of a protonated 4-(4-ethynylphenyl)-triazole functionalized quinoline drug model with ferriprotoporphyrin IX μ-oxo dimer shown in Figure 5 of the manuscript. Note no negative frequencies verifying a true minimum.

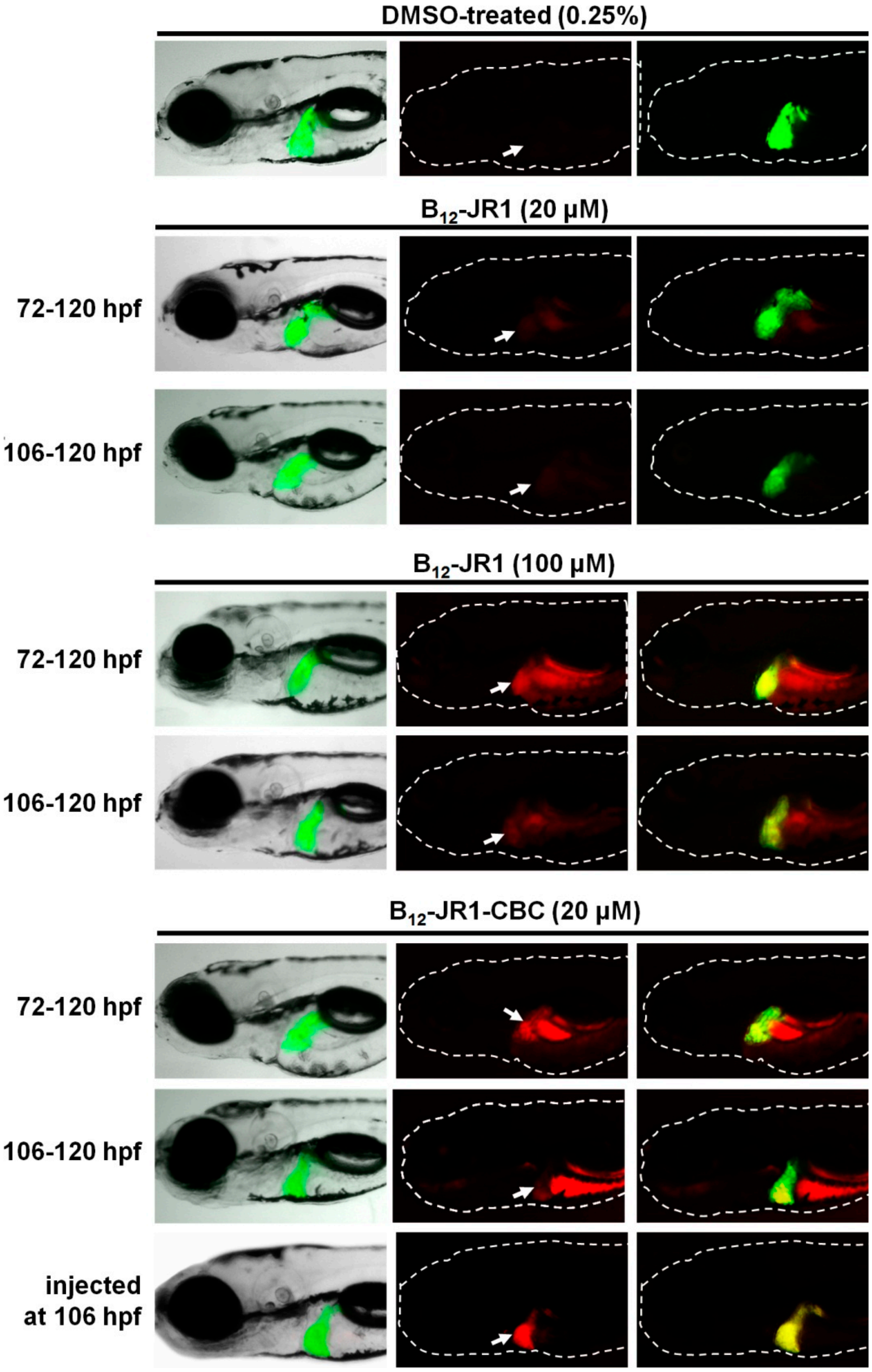

Figure S23. Biodistribution and accumulation of B12-JR1 and B12-JR1-CBC in the 120-hpf old transgenic Tg(fabp10:EGFP) zebrafish embryo with fluorescently labelled liver applied at different developmental stages.

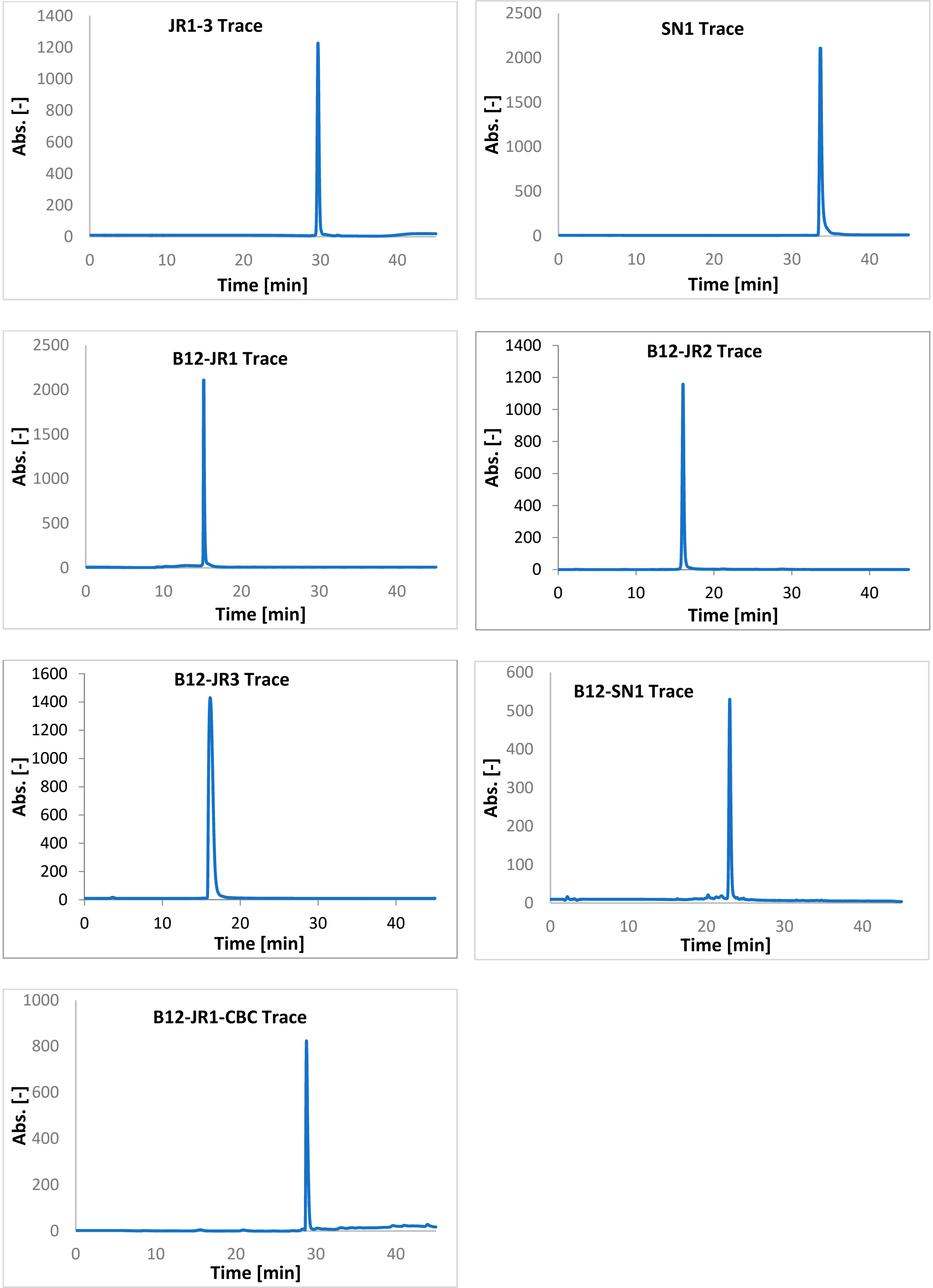

Figure S24. HPLC traces of molecules prepared in this study.
